# Emotional Processing in a Nap Paradigm: The Role of Sleep Stages in Memory and Reactivity

**DOI:** 10.1111/psyp.70414

**Published:** 2026-10-15

**Authors:** Costanza Colombo, Angie Baldassarri, Umberto Granziol, Jessica Ongaro, Lorenzo Viselli, Michele Ferrara, Aurora D'Atri, Nicola Cellini

**Affiliations:** ^1^ Department of General Psychology University of Padua Padua Italy; ^2^ Department of Biotechnological and Applied Clinical Sciences University of L'Aquila L'Aquila Italy

**Keywords:** emotional memory, emotional processing, emotional reactivity, REM sleep

## Abstract

It is widely known that sleep has a role in modulating emotional processing. However, while sleep facilitates the consolidation of emotional memories, its influence on emotional reactivity remains unclear. Most prior work has not fully addressed emotional reactivity, often relying solely on subjective or physiological measures, nor has it disentangled the contribution of different sleep stages. Here, using a between‐subjects design, we asked participants to complete two picture‐based tasks assessing emotional memory and emotional reactivity before and after a manipulation condition (nap, *N* = 40; wake, *N* = 40), and again 2 days later. Spontaneous variations in nap duration resulted in participants reaching REM sleep (REM group, *N* = 20) or not (No‐REM group, *N* = 20). Emotional reactivity, assessed using an emotional picture task consisting of 28 images with different emotional contents (negative and neutral), was measured through subjective ratings and physiological responses (phasic cardiac response and skin conductance response). Sleep preserved memory performance relative to wakefulness, but after 2 days, only negative images maintained a memory advantage in the Nap group; however, no differences emerged as a function of REM sleep occurrence during the nap. For emotional reactivity, subjective arousal decreased after sleep in the REM group, whereas valence ratings declined only after 2 days. Physiologically, heart rate habituation was greater in the No‐REM group. Together, these findings indicate that sleep fosters emotional memory consolidation while modulating reactivity in ways that diverge across subjective and physiological domains, with a decrease in subjective arousal ratings and an increase in phasic cardiac response. These findings, which should be interpreted in the context of the observed differences in participants' sleep architecture, highlight the complex role of sleep in emotional processing and underscore the need for standardized experimental paradigms.

## Impact Statement

Using a nap paradigm that naturally varies REM sleep occurrence, this study combines behavioral and psychophysiological measures to examine stage‐dependent effects of sleep on emotional processing. Sleep appears to exert a protective effect on emotional memory relative to wakefulness. Although our findings point to a role for REM sleep in emotional reactivity, the additional contributions of TST and N3% underscore the multifaceted nature of sleep‐related emotional processing.

## 1 Introduction

Emotions play a crucial role in our lives, guiding our behavior and shaping how we learn and remember. The way we recall an event depends not only on how we initially encoded it, but also on the consolidation process. This mechanism seems to be strongly modulated by emotions. In fact, while neutral events tend to be gradually forgotten, emotional memories tend to be preserved over time (LaBar and Cabeza 2006).

The role of sleep in the consolidation of declarative memories is well‐established (Marshall and Born 2007; Rasch and Born 2013), and this effect appears to extend to emotional information as well (Tempesta et al. 2018). Numerous studies have highlighted the beneficial impact of sleep on emotional memory consolidation, showing that a period of sleep enhances the retention of emotional memories more effectively than an equivalent period spent awake (Hu et al. 2006; Payne and Kensinger 2010), independent of stimuli valence (Davidson et al. 2021; Lipinska et al. 2019; Schäfer et al. 2020). More complex is determining whether this process can be ascribed to a specific sleep stage or rather reflects an interaction between non‐rapid eye movement (NREM) and rapid eye movement (REM) sleep stages. While NREM sleep is well known to be involved in declarative memory consolidation processes (Klinzing et al. 2019), REM sleep appears to provide the ideal condition for processing emotional information (Walker and Van Der Helm 2009). On a neurochemical level, REM sleep is characterized by a high concentration of acetylcholine (ACh), which plays a crucial role in facilitating the communication between the amygdala, the hippocampus, and the medial prefrontal cortex, involved in emotional memory processing and emotional valence regulation (Cabrera et al. 2024; Genzel et al. 2015; McGaugh 2004). In line with this, several studies using the split‐night paradigm have highlighted a preferential effect of late‐night sleep on emotional memories, suggesting that the retention of emotional material is better during REM‐rich sleep compared to early‐night NREM‐rich sleep (Groch et al. 2015; Wagner et al. 2001). Similar results have been obtained using the nap paradigm (Alger and Payne 2016; Nishida et al. 2009), indicating a positive correlation between the amount of REM sleep and the consolidation of negative images. However, the idea that different sleep stages contribute to the processing of different types of memories (Rasch and Born 2013) is opposed by the hypothesis that the succession of both NREM and REM states is essential for memory processing (Giuditta 2014).

As a part of the emotional processing, emotional reactivity, the psychophysiological response to an emotional stimulus, is relevant in promoting memory consolidation. Several studies have demonstrated that stimuli with high arousal are better consolidated than stimuli with lower arousal (Kensinger 2004; Mickley Steinmetz et al. 2010). Research investigating the contribution of sleep to emotional reactivity has employed paradigms involving exposure to emotional material (e.g., images), using subjective and physiological measures (i.e., skin conductance response (SCR), phasic cardiac response (PCR)), and comparing pre‐ and post‐sleep reactivity to the same set of stimuli (Lipinska et al. 2022). Studies on reactivity show mixed results, not clarifying whether sleep leads to depotentiation (Bolinger et al. 2019; Cunningham, Crowell, et al. 2014; Pace‐Schott et al. 2011), maintenance (Baran et al. 2012), or enhancement (Wagner 2002) of emotional reactivity.

Considering the role of sleep stages, several studies have shown that REM sleep is associated with a depotentiation in emotional reactivity, evident in both subjective and physiological responses (Cunningham, Crowell, et al. 2014; Cunningham and Payne 2017; Gujar et al. 2011; Viselli et al. 2025). This effect may be attributed to neurochemical changes that occur during REM sleep. In particular, high cholinergic modulation and the almost complete absence of adrenergic activity favor the remapping of emotional memories, contributing to their reprocessing and depotentiation (Cabrera et al. 2024).

The “Sleep to forget, sleep to remember” (SFSR) hypothesis (Walker and Van Der Helm 2009) offers an integrative framework that accounts for both memory consolidation and emotional reactivity processes, suggesting that, considering the neurochemical, functional, and electrical changes occurring during REM sleep, this stage represents the ideal scenario to allow two processes: on the one hand, the stabilization of the informational core of an emotional event, and on the other hand, the depotentiation of the autonomic activation acquired at the time of learning. In other words, REM sleep should help to maintain the memory of an event, while its emotional tone should be reduced.

However, the “Emotional Salience Consolidation” (ESC) hypothesis proposes that REM sleep may act as a protector of emotional reactivity, preserving (Baran et al. 2012) or enhancing it (Lara‐Carrasco et al. 2009; Wagner 2002; Werner et al. 2021). This divergence in findings may be due to differences in methodologies, such as the use of different measures to assess emotional reactivity (i.e., subjective or physiological measures), variations in waking control conditions, or differences in the emotional stimuli presented (Lipinska et al. 2022).

In this complex scenario, the present study aimed to analyze the contribution of an afternoon nap to emotional processing using a multimodal approach that combines behavioral and psychophysiological measures. Emotional processing was examined by separately assessing the emotional memory and the reactivity components, both before and after a manipulation condition (i.e., nap or wake), as well as 2 days later. To observe the specific contribution of REM sleep to emotional processing, nap duration and structure were naturally determined, with participants either reaching REM sleep or not.

Based on the SFSR model, we tested several research hypotheses. First, we expected REM sleep to contribute to the attenuation of emotional reactivity, both physiologically and subjectively. Second, we expected REM sleep to protect the emotional information. In particular, regarding emotional memory, the Nap group (i.e., the group that spent the period sleeping, regardless of the sleep stage reached) was expected to show better memory consolidation scores than the Wake group, both for neutral and negative images. Additionally, the REM group (i.e., the participants who reached the REM sleep stage) was expected to show better memory performance for the negative images compared to the No‐REM group (i.e., the participants who did not reach the REM sleep stage). Regarding emotional reactivity, we expected a reduction over time in both physiological and subjective responses in all groups, with a greater reduction in the REM group compared to the No‐REM group, as well as between the Nap and Wake groups, after the manipulation condition. In the delayed recognition test (i.e., 2 days after), no differences were expected between all groups, as all participants had experienced multiple REM cycles.

## 2 Materials and Methods

### 2.1 Participants

Eighty‐nine participants between the ages of 19 and 35 years were recruited for the study on a voluntary basis. Of these, nine participants were excluded from the analyses: six were excluded because they were unable to fall asleep, two because they slept for less than 15 min, and one was excluded due to compromised polysomnographic recording, which made it impossible to determine whether the participant had fallen asleep.

The final sample consisted of 80 participants (39 F; mean age = 23.4; SD = 2.5), with 40 participants assigned to the Nap group and 40 to the Wake group. Participants in the Nap group were further divided into No‐REM (*N* = 20) and REM (*N* = 20) subgroups based on whether they reached the REM sleep stage. No power analysis was conducted to estimate the final sample size since the effects of REM sleep on emotional processing present in the literature are still very mixed and contrasting.

No formal exclusion criteria were applied. However, we recruited participants without any diagnosis of psychiatric disorder or blood phobia, which were assessed through direct questioning. In addition, for the Nap group, only individuals who were inclined to take afternoon naps were preferred. This resulted in a non‐random assignment to the experimental groups.

The study protocol was approved by the Ethics Committee of the Department of General Psychology of the University of Padova.

### 2.2 Experimental Tasks

The experimental design provided each group with two tasks: the first assessing emotional reactivity and the second assessing emotional memory, always administered in the same order (Figure 1a,b). Different image sets were used for the emotional reactivity and picture recognition tasks to minimize potential interference effects between the recognition of a stimulus and the emotional response to it. In addition, separate stimulus sets allowed the tasks to be optimized for their specific methodological requirements, including physiological recording constraints (e.g., habituation and response timing) and memory task difficulty.

**FIGURE 1:**
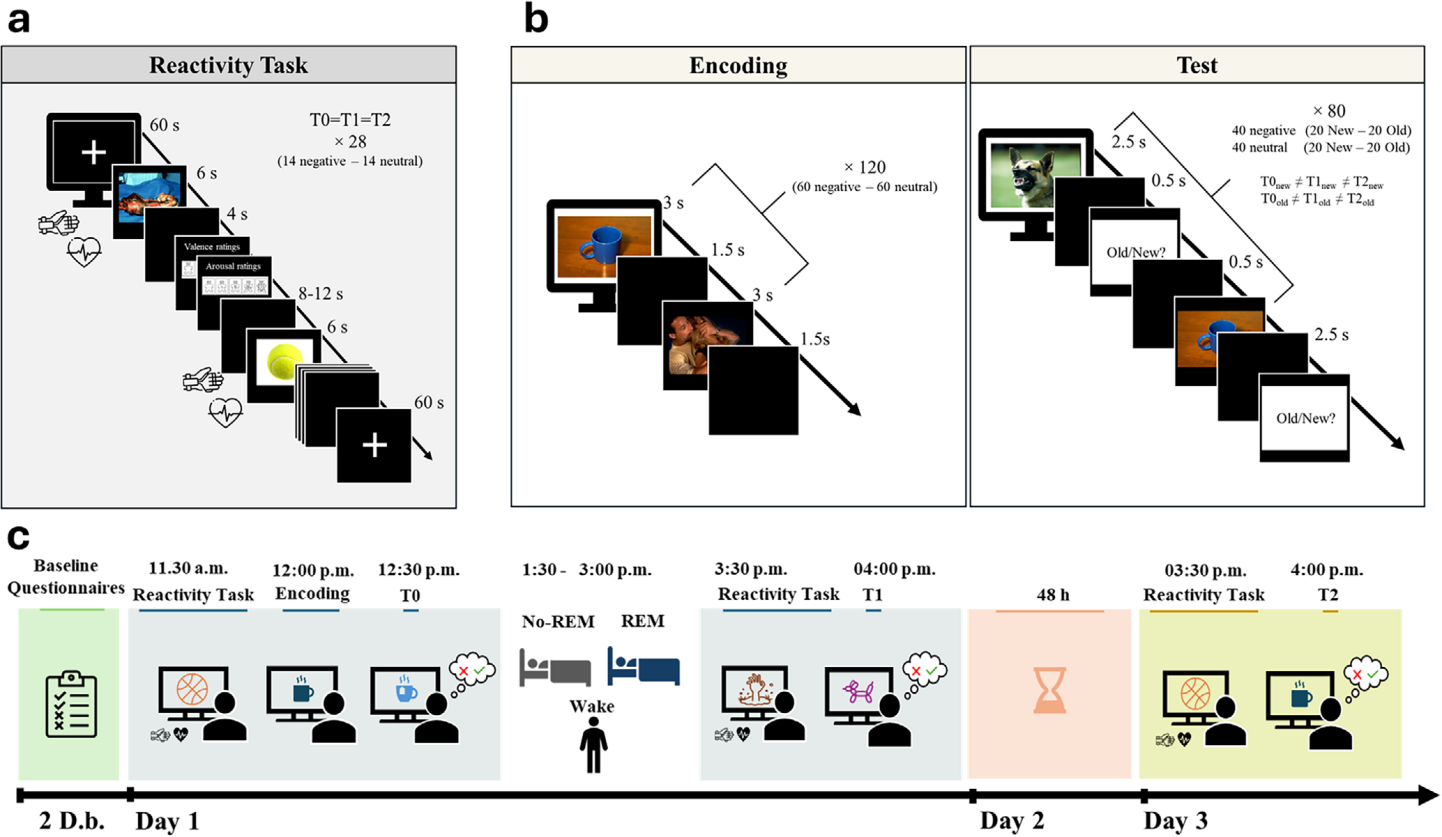
Tasks and experimental procedure. (a) Schematic representation of the Reactivity Task. The task consisted of the exposure to 28 pictures (14 negative and 14 neutral), randomly shown during each experimental session (T0, T1, T2). The pictures were displayed for 6 s, followed by a 4‐s ISI. Afterwards, participants were required to provide subjective ratings for arousal and valence using the Self‐Assessment Manikin (SAM) with ITI varying between 8 and 12 s. Baseline measurements were collected for 60 s at the beginning and end of the task. (b) Schematic representation of the Picture Recognition Task. *Encoding*. During the encoding phase, 120 pictures (60 negative and 60 neutral) were randomly presented. Each picture was displayed for 3 s with an ISI of 1.5 s. *Test*. During the test phase (T0, T1, T2), 40 old pictures (20 negative and 20 neutral) were presented alongside 40 new pictures in each experimental session. Each picture was displayed for 2.5 s with an ISI of 0.5 s. Both old and new pictures were randomly shown once per experimental session (T0 ≠ T1 ≠ T2), such that pictures presented in one test phase, whether old or new, did not reappear in subsequent test phases. This ensured that participants were tested on each picture only once. (c) Experimental procedure. Two days before the experiment, participants filled out screening questionnaires. On day one, they arrived at the laboratory at 11:30 a.m. to perform the reactivity task. Following that, the encoding phase of the Picture Recognition Task started at 12:00 p.m. After 15 min, they completed the test phase. Participants who slept in the laboratory were prepared for the polysomnography setup, while the Wake group was free to leave the laboratory. In the afternoon of Day 1 (3:30 p.m.), all participants performed the tasks completed in the morning, as well as 2 days later (Day 3). D.b. = Days before.

#### 2.2.1 Reactivity Task

To observe changes in emotional reactivity, we used an exposure picture task (Reactivity Task; Figure 1a).

Fifty‐six pictures were selected: 28 depicting high‐arousal negative content (e.g., mutilations, injuries) selected from the International Affective Picture System (IAPS) (Bradley and Lang 2007), and 28 depicting neutral content (e.g., sport equipment) selected from the Mnemonic Similarity Task (Stark et al. 2019). The list of stimuli used for this task can be found in Table S18. Four versions of the task were created, each containing 14 negative and 14 neutral pictures, with similar evaluations for subjective arousal and valence (see Table S18 for each set's mean ratings). Each participant was assigned to one of the four sets and tested on it during each experimental session. Image sets were counterbalanced for the Nap and Wake groups; however, counterbalancing for the No‐REM and REM groups was not practicable, as this classification was performed post hoc based on sleep architecture, making a priori task assignment impossible. The distribution of picture‐set assignments across groups was evaluated using Fisher's exact test, which did not reveal a significant difference in set allocation between groups (*p* = 0.445; Table S20). The pictures were presented in a random order and remained the same across sessions. Each picture was displayed for 6 s, followed by a 4‐s interstimulus interval (ISI), after which participants rated their perceived valence and arousal level using the Self‐Assessment Manikin (SAM) (Bradley and Lang 1994), with no time limit for responses. Following the subjective ratings, the intertrial interval (ITI) varied between 8 and 12 s. During the task, electrodermal activity (EDA) and electrocardiogram (ECG) were recorded. A 60‐s baseline period was recorded at the beginning and end of the task, during which a white cross was displayed at the centre of the screen.

#### 2.2.2 Picture Recognition Task

A Picture Recognition Task was used to assess emotional memory. This task was designed to investigate the level of retention of the encoded material (Figure 1b).

The task consisted of 240 pictures, selected both from the IAPS and the Nencki Affective Picture System (NAPS) (Marchewka et al. 2014). The list of stimuli used for this task can be found in Table S19. In the encoding phase, 120 pictures with varying emotional content (60 neutral and 60 negative) were shown. Each picture was displayed for 3 s, followed by a 1.5‐s ISI. During the immediate test phase (T0), the second test phase (T1), and the test phase conducted 48 h later (T2), 80 pictures were presented in each session. Of these, 40 (20 negative and 20 neutral) were shown during the encoding phase, while the other 40 (20 negative and 20 neutral) were new. The new and old pictures presented during each test phase were unique to that session and were not repeated in subsequent tests, ensuring that each participant was tested on each picture only once. Each picture was presented for 2.5 s with an ISI of 0.5 s, after which participants were required to respond. The participant's task was to recognize whether the presented picture was “old,” meaning it had been shown during the encoding phase, or new. Participants were given unlimited time to respond.

The entire protocol was conducted using the software PsychoPy 2 (Peirce et al. 2019).

### 2.3 Experimental Procedure

The experimental procedure is summarized in Figure 1c.

As mentioned above, participants were divided into three groups: the Wake group, which did not take a nap in the laboratory, and the REM and No‐REM groups, which slept in the laboratory with a 90‐min window of opportunity. The REM and No‐REM groups were categorized afterwards based on sleep stage scoring. Tasks were performed at three different time points: before the experimental condition (i.e., nap or wakefulness period; T0), immediately after (T1), and 48 h later (T2).

Two days before participating in the study, participants completed online questionnaires aimed at investigating basic demographic information and aspects related to sleep, circadian preference, severity of depressive and anxiety symptoms, and severity of blood phobia. Specifically, the assessed aspects included the severity of insomnia symptoms using the Insomnia Severity Index (ISI) (Bastien 2001; Castronovo et al. 2016), daytime sleepiness propensity measured with the Epworth Sleepiness Scale (ESS) (Johns 1991; Vignatelli et al. 2003), self‐reported sleep quality assessed using the Pittsburgh Sleep Quality Index (PSQI) (Buysse et al. 1989; Curcio et al. 2013), severity of anxiety, depression, and stress symptoms using the Depression, Anxiety, and Stress Scale‐21 (DASS‐21) (Bottesi et al. 2015; Henry and Crawford 2005), circadian preference measured with the reduced version of the Morningness‐Eveningness Questionnaire (MEQ‐r) (Adan and Almirall 1991; Natale et al. 2006), and presence of blood phobia assessed with the Mutilation Questionnaire (MQ) (Kleinknecht and Thorndike 1990).

The experimental procedure took place at the Sleep Psychophysiology Laboratory of the Department of General Psychology at the University of Padova. Participants arrived at the laboratory at 11:15 a.m. and were seated in a comfortable room, where informed consent was obtained.

The Reactivity Task started at 11:30 a.m. and lasted approximately 15 min. After applying electrodes to measure ECG and EDA, each participant was instructed on the task. Upon completion of the reactivity task, a 10‐min break followed before starting the encoding phase of the Picture Recognition Task, which began at 12:00 p.m. Participants were instructed to pay attention to the pictures presented, as they would be shown again along with new stimuli in subsequent phases. Fifteen minutes after the learning phase, the immediate test (T0) was conducted. At the end of the Picture Recognition Task, participants in the Wake group were dismissed. They were reminded not to consume caffeine in the 2 h preceding the test (T1) and were instructed to return to the laboratory at 3:30 p.m.

Participants in the Nap group were prepared for the application of the polysomnography (PSG). After the PSG setup, participants were provided with earplugs to improve acoustic isolation. The lights were turned off around 1:30 p.m.

At 3:30 p.m., all groups underwent the Reactivity Task and the test phase (T1) of the Picture Recognition Task, following the same procedures as in the morning. Similarly, 2 days later, at 3:30 p.m., participants from both groups returned to the laboratory to perform the Reactivity Task and the test phase (T2) of the Picture Recognition Task.

At the end of the experiment, a debriefing was conducted.

### 2.4 Psychophysiological and Behavioral Measures

As general procedure for managing the artifacts, all signals were visually inspected. Where motion artifacts were present, their impact on signal quality was assessed. Given that both the PCR and SCR analyses focused on the 6‐s image exposure window (as well as the 2‐s pre‐stimulus period for the PCR), the signals included in the sample did not exhibit motion artifacts that compromised signal integrity.

#### 2.4.1 EDA

The EDA signal was acquired using the BioSignal Plux system (PluxBiosignal. Lisbon, PT) with a sampling rate of 500 Hz and downsampled to 10 Hz.

The signal was processed using the Matlab toolbox Ledalab (www.ledalab.de). The pre‐processing consisted of an adaptive smoothing and a second‐order low‐pass Butterworth filter with a cut‐off frequency of 1 Hz. Each signal was analyzed using the Continuous Decomposition Analysis (CDA) (Benedek and Kaernbach 2010), which allows the distinction of the signal between phasic and tonic activity.

The time window used to evaluate the skin conductance response (SCR, μS) was set between 0 and 6 s, corresponding to the presentation time of each picture.

Based on the output of CDA analysis, we selected the CDA.SCR parameter, as it reflects the average phasic driver activity within the stimulus presentation window. For each participant and session, mean CDA.SCR values were then calculated for both categories of images.

Participants who exhibited significant skin conductance responses for less than 30% of the stimuli at T0 were identified as non‐responders and excluded from the final analyses. Non‐responders were identified using the CDA.nSCR parameter from Ledalab, which represents the number of responses exceeding the significance threshold (set at 0.01 μS) for each image.

#### 2.4.2 ECG

The ECG signal was recorded using the same BioSignal Plux System (PluxBiosignal. Lisbon, PT) with a sampling frequency of 500 Hz. The R‐R intervals were detected from the ECG signal using the open‐source software EDFBrowser (https://www.teuniz.net/edfbrowser/). Cardiac reactivity during stimulus presentation was analyzed using the Matlab toolbox Kardia (Perakakis et al. 2010). The baseline was defined as the 2 s before each picture's presentation and was compared to the 6 s during which each picture was shown. The Phasic Cardiac Response (PCR) grand average was calculated by first obtaining each participant's average heart rate (HR) during the 6 s of stimulus presentation across all images, categorized by image type (negative and neutral), and then by subtracting the average baseline value from the HR in each second (i.e., HR_t_—HR_baseline_). Therefore, we obtained the mean HR change from the baseline for each second of stimulus presentation (∆PCR), with negative values indicating HR deceleration from the baseline.

#### 2.4.3 PSG

PSG recording included 2 electrodes for HR monitoring, 2 electrodes for electrooculogram (EOG) measurement, 2 electrodes for chin electromyogram (EMG) measurement, and electroencephalography (EEG) recording with 8 scalp electrodes (F3, F4, C3, C4, P3, P4, O1, O2) positioned according to the international 10–20 system, along with two electrodes on the mastoids (M1, M2). Impedances were kept below 5kΩ. The PSG signals were acquired using the V‐Amp 16 amplifier and the BrainVision Recorder software (Brain Products GmbH 2021) at a sampling rate of 250 Hz, referencing online to Cz. Afterwards, data were filtered (high‐pass and low‐pass filters at 0.3 and 35 Hz, respectively; notch filter at 50 Hz) and re‐referenced to contralateral mastoids. Sleep data were visually scored by a trained experimenter (NC) within 30‐s epochs following the American Academy of Sleep Medicine guidelines (Iber et al. 2007). To be included in the REM group, a participant had to reach the REM sleep stage for more than 1 min, following the criteria established by McDevitt et al. (2014).

#### 2.4.4 Behavioral Measures

For the subjective ratings of arousal and valence associated with the pictures in the Reactivity Task, single‐trial responses for negative and neutral pictures were considered for each participant and each experimental session.

Memory performance was assessed using the d‐prime index. The d‐prime is a general accuracy index, derived from the difference between the z‐scores of Hit Rate and False Alarm Rate (Hautus et al. 2021). Hit Rate corresponds to the proportion of old pictures correctly identified as such, and the False Alarm Rate corresponds to the proportion of new pictures mistakenly identified as “already seen.” However, this calculation does not work when the Hit Rate is equal to 1 or the False Alarm Rate is equal to 0. In such cases, the value of Hit Rate was adjusted by dividing the number of observations minus 1 by the total number of observations, as $\left(N-1\right)/N$, and False Alarm Rate resulted from dividing 1 by the total number of observations, as 1/N.

### 2.5 Statistical Analyses

Measurements at T2 of three participants (one for each group) are missing because they did not return to the laboratory on the second experimental day.

Demographic, psychological, and sleep variables were described using either mean and standard deviation or median and interquartile range, depending on the statistical test used. Demographic and psychological variables were compared between groups using one‐way ANOVA tests, with Group (No‐REM, REM, Wake) as a between‐subjects factor, Kruskal‐Wallis tests (*H*) or chi‐squared tests (*χ* ^2^), whereas sleep parameters were compared between groups (No‐REM, REM) using *t*‐tests (*t*) and Wilcoxon tests (*W*). Post hoc tests were conducted using *t*‐tests and corrected with Holm's method. For each test, the measure of the effect size reported was either eta‐squared for the ANOVA (*η* ^2^) and the Kruskal‐Wallis tests (*η* ^2^[*H*]), the Cohen's d (*d*) for the *t*‐tests, the Cramer's V (*V*) for chi‐square tests, and the Wilcoxon's r (*r*) for the Wilcoxon tests.

For the behavioral and psychophysiological data, we conducted linear or generalized mixed models, depending on the type and distribution of the data. Three different models were run for each dependent variable: one model on T0, to assess baseline differences, and two separate models for each image type (i.e., negative and neutral), to avoid three‐way interactions, which are often difficult to interpret in mixed models.

The models on T0 were conducted including Type of image (Negative, Neutral) as a within‐subjects fixed effect, and Group (No‐REM, REM, Wake) as a between‐subjects fixed effect, to check for possible group differences before the experimental manipulation and to confirm the presence of differences between negative and neutral images. Results of this model for each dependent variable can be found in the Supporting Information S1.

The models on negative and neutral images were conducted including Session (T0, T1, T2) as a within‐subjects fixed effect and Group (No‐REM, REM, Wake) as a between‐subjects fixed effect.

Since this was not designed as an exploratory study, we decided to set a priori contrasts (Schad et al. 2020) to test specific hypotheses established before data analysis, thereby reducing the risk of spurious findings associated with the high number of multiple comparisons that could have arisen. For the models on T0, we applied Helmert contrast to the Group factor (No‐REM, REM, Wake). The Helmert contrast yields two comparisons: the first captures the difference between the REM and No‐REM groups (i.e., REM−NoREM), and the second captures the difference between the Wake group and the mean of the REM and No‐REM groups (i.e., $\text{Wake}-\frac{\mathrm{REM}+\mathrm{No}-\mathrm{REM}}{2}$). In this way, we tested for differences within the Nap group (i.e., REM and No‐REM) and between the Wake and the Nap group, as no specific hypotheses were proposed regarding differences between the Wake group and either the REM or No‐REM groups (see Introduction). For the models on negative and neutral images, we applied Helmert contrasts to the Group factor, as explained above, and Repeated contrasts to the Session factor (T0, T1, T2). Repeated contrasts compare adjacent factor levels with each other; in our case, this results in two comparisons: the first is the difference between T1 and T0 (i.e., T1−T0), and the second is the difference between T2 and T1 (i.e., T2−T1).

To assess memory performance, linear mixed models were conducted on d‐prime using the lme4 package (Bates et al. 2015) in R. Both the model on T0 and the models on negative and neutral images included participants' ID as a random intercept to account for individual differences.

To investigate changes in subjective arousal and valence, we conducted separate ordinal regression mixed models, specifically cumulative models with a logit link function (Agresti 2010; Gambarota and Altoè 2024; McCullagh 1980) with the ordinal package (Christensen 2023) in R. Both the model on T0 and the models on negative and neutral images included participants' ID as a random intercept.

For the SCR analysis, two participants, one from the No‐REM and one from the REM group, were excluded from the final analyses because they responded to less than 30% of the stimuli at T0 and were therefore classified as non‐responders. We also excluded from the SCR analysis one participant at T1 from the Wake group and one at T2 from the REM group due to compromised skin conductance recording.

Changes in SCR were assessed using a generalized mixed model with a Gamma distribution and a log link function using the lme4 package in R. Both the model on T0 and the models on negative and neutral images included participants' ID as a random intercept.

To assess changes in ∆PCR we conducted linear mixed models using the lme4 package in R. Both the model on T0 and the models on negative and neutral images included participants' ID as a random intercept. In addition, models on negative and neutral images included the time of image presentation (6 s) nested within the Session.

As a measure of effect size, standardized coefficients (β*) were computed for all models using the parameters package (Lüdecke et al. 2020) in R, except for the ordinal regression mixed models for which odds ratios (OR) were used instead.

Outliers were identified using Cook's distance method, with the threshold set at 0.5. Analyses were conducted both with and without these observations to assess their influence; when results were unaffected, models including outliers were retained; otherwise, we reported both models.

Given the variability in total sleep time (TST), and in the percentage of time spent in N3 (N3%) and REM sleep (REM%) within the Nap group, analyses were conducted to examine their impact on emotional processing. Linear or generalized mixed models were applied, using only the Nap group and the T0 and T1 sessions as predictors, focusing exclusively on negative images. The variables included were the same as those used in the previous analyses, except for subjective ratings of Arousal and Valence, for which the mean scores for negative images were considered (see Supporting Information S1, Section 6 and 7).

Statistical analyses were conducted using RStudio (R Core Team 2023), with *p* < 0.05.

## 3 Results

### 3.1 Demographic Variables

From Table 1 we can observe a difference in PSQI results between groups, indicating better sleep quality in both the REM and No‐REM groups compared to the Wake group. However, this difference was not statistically significant (*p*_adjusted_ = 0.257). The three groups differed in their ESS results (*p*_adjusted_ < 0.001). Particularly, post hoc comparisons showed that the REM and the No‐REM groups exhibited higher daytime sleepiness than the Wake group (REM compared to Wake: *t*_34_ = 3.56, *p*_adjusted_ = 0.002, *d* = 1.03; No‐REM compared to Wake: *t*_41_ = 5.40, *p*_adjusted_ = < 0.001, *d* = 1.44).

**TABLE 1:** Mean and standard deviation (SD) or median and interquartile range (IQR) of demographic and psychological variables in No‐REM, REM, and Wake groups.

Measures		Group			Test	*p*	Effect size
		No‐REM (*N* = 20)	REM (*N* = 20)	WAKE (*N* = 40)			
Age (years)	*M* (IQR)	22.7 (2.6)	22.2 (2.5)	23.5 (2.3)	*χ* ^2^ = 4.73	0.941	*η* ^2^ [*H*] = 0.03
Gender^a^	M/F	11/9	10/9	19/21	*χ* ^2^ = 0.34	> 0.999	*V* = 0.07
DASS‐21 (global score)	*M* (IQR)	16.5 (16.5)	14.0 (10.2)	15.5 (14)	*χ* ^2^ = 0.25	> 0.999	*η* ^2^ [*H*] = −0.02
Depression (DASS‐21)	*M* (IQR)	5.0 (8)	4.5 (5.2)	5.0 (7)	*χ* ^2^ = 0.46	> 0.999	*η* ^2^ [*H*] = −0.02
Anxiety (DASS‐21)	*M* (IQR)	2.0 (3.5)	3.0 (3)	2.0 (3.5)	*χ* ^2^ = 0.26	> 0.999	*η* ^2^ [*H*] = −0.02
Stress (DASS‐21)	*M* (IQR)	7.5 (8)	6.5 (4.2)	7.0 (7)	*χ* ^2^ = 0.79	> 0.999	*η* ^2^ [*H*] = −0.02
PSQI	*M* (IQR)	4.0 (1.5)	4.0 (3.2)	6.0 (3)	*χ* ^2^ = 7.50	0.257	*η* ^2^ [*H*] = 0.07
ISI	*M* (SD)	5.5 (4.5)	6.0 (3.5)	7.0 (6.2)	*χ* ^2^ = 3.76	> 0.999	*η* ^2^ [*H*] = 0.02
ESS	*M* (SD)	9.2 (2.9)	8.2 (3.6)	4.8 (3.1)	*F* = 15.23	< 0.001	*η* ^2^ = 0.28
MQ	*M* (IQR)	8.0 (4.5)	7.5 (6.2)	8.5 (6.0)		> 0.999	*η* ^2^ [*H*] = 0.001
Circadian type (MEQ‐r)	I/M/E^b^	16/2/2	13/2/5	22/7/11	*χ* ^2^ = 2.09^c^	> 0.999	*V* = 0.16

*Note:* *p*‐values in the table are adjusted for multiple comparisons. Test statistics, *p*‐values, and effect sizes of between‐groups comparisons are reported.

Abbreviations: DASS‐21, Depression, Anxiety, and Stress Scale—21 Items; ESS, Epworth Sleepiness Scale; ISI, Insomnia Severity Index; MEQ‐r, Morningness–Eveningness Questionnaire reduced version; MQ, Mutilation Questionnaire; PSQI, Pittsburgh Sleep Quality Index.

^a^ One participant (REM Group) indicated an unspecified gender.

^b^ Intermediate‐type, Morning‐type, and Evening‐type.

^c^ Exact Fisher's test performed.

### 3.2 Sleep Parameters

The REM and No‐REM groups showed differences in all sleep parameters except for the time spent in bed and the time spent in N1 stage. After correcting *p*‐values for multiple comparisons, SOL (*p*_adjusted_ = 0.143) and N2 (*p*_adjusted_ = 0.143) did not show significant differences between groups. The REM group slept more than the No‐REM group (TST), with less time spent awake (WASO). The No‐REM group spent most of its nap in lighter sleep stages, such as N1 and N2, compared to the REM group, which had a more even distribution across all sleep stages (see Table 2).

**TABLE 2:** Mean and standard deviation (SD) or median and interquartile range (IQR) of sleep parameters in No‐REM and REM groups.

Sleep parameters		Group		Test	*p*	Effect size
		No‐REM (*N* = 20)	REM (*N* = 20)			
TIB (min)	*M* (SD)	90.3 (16.6)	98.7 (8.6)	*t* = −1.99	0.144	*d* = −0.63
TST (min)	*M* (SD)	44.7 (19.0)	83.4 (14.7)	*t* = −7.22	< 0.001	*d* = −2.28
SE (%)	*M* (SD)	51.2 (23.8)	84.1 (10.9)	*t* = −5.62	< 0.001	*d* = −1.78
SOL (min)	*M* (IQR)	7.5 (8.6)	5.0 (3.6)	*W* = 273.50	0.143	*r* = 0.32
WASO (min)	*M* (IQR)	40.5 (38.8)	7.2 (11.1)	*W* = 330.00	0.003	*r* = 0.56
N1 (min)	*M* (SD)	10.0 (3.9)	9.5 (3.7)	*t* = 0.44	0.663	*d* = 0.14
N2 (min)	*M* (IQR)	22.7 (22.6)	32.5 (17.3)	*W* = 122.00	0.143	*r* = 0.33
N3 (min)	*M* (IQR)	1.5 (12.8)	25.2 (22.3)	*W* = 61.00	0.001	*r* = 0.60
N2 + N3 (min)	*M* (SD)	34.7 (18.4)	58.5 (12.6)	*t* = −4.77	< 0.001	*d* = −1.51
REM (min)	*M* (SD)	—	15.3 (7.6)	—	—	—
N1 (%)	*M* (IQR)	24.9 (15.4)	10.0 (8.4)	*W* = 325.00	0.003	*r* = 0.54
N2 (%)	*M* (IQR)	66.2 (19.7)	46.6 (19.8)	*W* = 331.00	0.002	*r* = 0.56
N3 (%)	*M* (IQR)	5.3 (20.2)	27.8 (22.6)	*W* = 101.50	0.037	*r* = 0.43
REM (%)	*M* (SD)	—	17.7 (7.2)	—	—	—

*Note:* *p*‐values in the table are adjusted for multiple comparisons. Test statistics, *p* values, and effect sizes of between‐groups comparisons are reported.

Abbreviations: SE, sleep efficiency; SOL, sleep‐onset latency; TIB, time in bed; TST, total sleep time; WASO, wake after sleep onset.

### 3.3 Memory Performance

The linear mixed model conducted on d‐prime to assess memory performance for negative images, with Session and Group as fixed effects, and subjects as random intercept, revealed lower memory performance in the Wake group compared to the Nap group (*β* = −0.08, SE = 0.03, *t* = −2.32, *p* = 0.023, β* = −0.12), as shown in Figure 2a. Additionally, the results indicated a decline in memory performance from T0 to T1 (*β* = −0.38, SE = 0.07, *t* = −5.37, *p* < 0.001, β* = −0.59) and from T1 to T2 (*β* = −0.36, SE = 0.07, *t* = −4.97, *p* < 0.001, β* = −0.55), regardless of group. Full model results can be found in Table S2.

**FIGURE 2:**
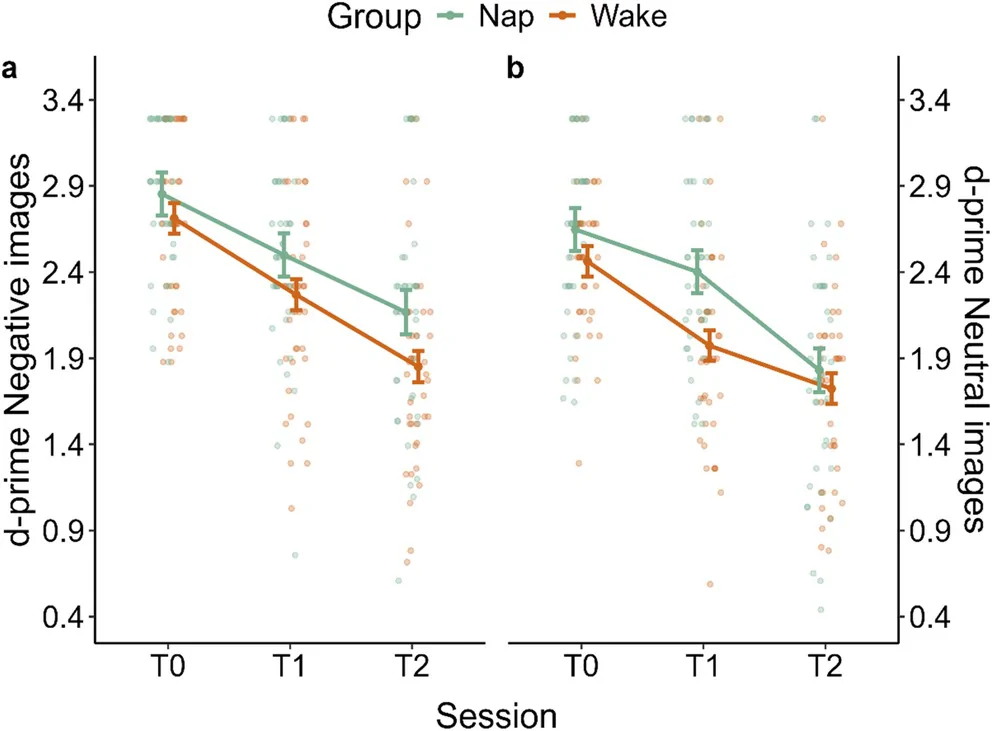
Memory performance for negative and neutral images. (a) D‐prime performance in the Wake and Nap groups for negative images across the three sessions. (b) D‐prime performance in the Wake and Nap groups for neutral images across the three sessions. Bigger dots represent the estimated mean by the model. Smaller dots represent individual subjects' values. Error bars represent the standard error estimated by the model.

The model conducted on d‐prime to assess memory performance for neutral images, with Session and Group as fixed effects, and subjects as random intercept, similarly showed lower memory performance in the Wake group compared to the Nap group (*β* = −0.08, SE = 0.04, *t* = −2.24, *p* = 0.028, β* = −0.12). Memory performance also declined from T0 to T1 (*β* = −0.33, SE = 0.06, *t* = −5.63, *p* < 0.001, β* = −0.50) and from T1 to T2 (*β* = −0.46, SE = 0.06, *t* = −7.86, *p* < 0.001, β* = −0.71), similarly to the trend observed for negative images. Moreover, a greater difference from T0 to T1 was observed in the Wake group than in the Nap group, with the Wake group showing a more pronounced memory decline from T0 to T1 than the Nap group (*β* = −0.08, SE = 0.04, *t* = −2.21, *p* = 0.028, β* = −0.12). The two groups also differed from T1 to T2, with a greater change observed in the Nap group (*β* = 0.11, SE = 0.04, *t* = 2.88, *p* = 0.005, β* = 0.16), indicating that the gap between the two groups diminished during this time interval (see Figure 2b). Full model results can be found in Table S3.

### 3.4 Emotional Reactivity

#### 3.4.1 Arousal

The cumulative link model conducted on Arousal to assess changes in subjective arousal for negative images, with Group and Session as fixed effects, and subjects as random intercept, revealed a difference between T1 and T0, indicating lower arousal scores at T1 compared to T0 for negative images (i.e., negative images were evaluated as less arousing at T1 than at T0) (log‐odds = −0.53, SE = 0.08, *z* = −6.44, *p* < 0.001, OR = 0.59). Moreover, a difference between REM and No‐REM groups across T0 and T1 sessions was found, indicating that the decrease in arousal level is greater in the REM group compared to the No‐REM group (log‐odds = −0.31, SE = 0.11, *z* = −2.86, *p* = 0.004, OR = 0.73) (Figure 3a). Additionally, a difference between the Nap and Wake groups across T0 and T1 sessions was found, indicating that the decrease in arousal level from T0 to T1 is greater in the Nap group compared to the Wake group (log‐odds = 0.17, SE = 0.05, *z* = 3.26, *p* = 0.001, OR = 1.18) (Figure 3b). Full model results can be found in Table S5.

**FIGURE 3:**
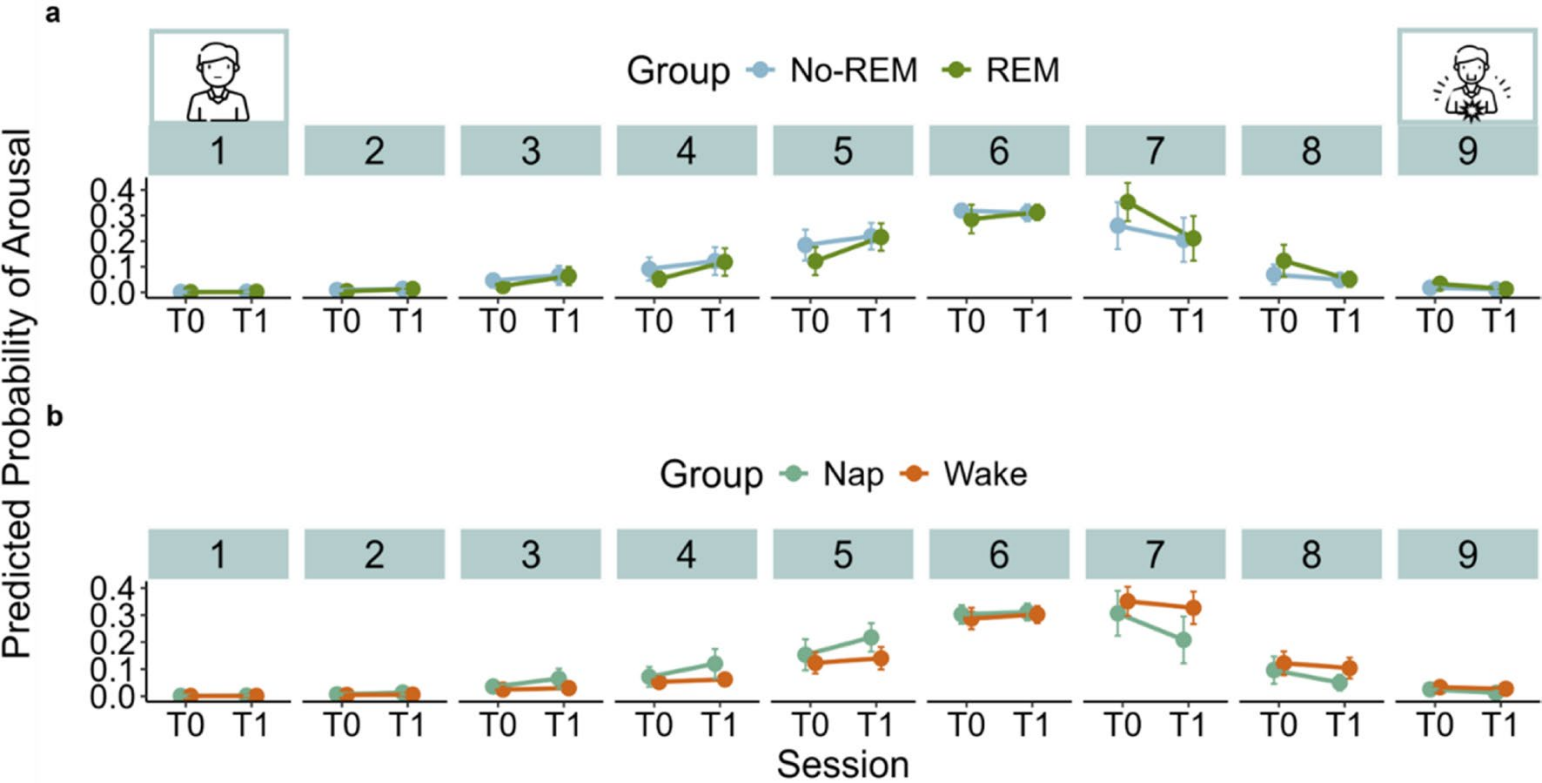
Predicted probability of Arousal for negative images across T0 and T1. (a) Comparison between the No‐REM and REM groups. (b) Comparison between the Nap and Wake groups. Each dot represents the mean probability estimated by the model for each ordinal level. Error bars represent the standard error estimated by the model. Numbers from 1 to 9 above the plot indicate ordinal responses on the Self‐Assessment Manikin, with 1 corresponding to low arousal levels and 9 to high arousal levels.

The cumulative link model conducted on Arousal to assess changes in subjective arousal for neutral images, with Group and Session as fixed effects, and subjects as random intercept, revealed a difference between T1 and T0, indicating that the neutral images were rated with high scores at T0 compared to T1 (i.e., neutral images were evaluated as less arousing at T1 compared to T0) (log‐odds = −0.58, SE = 0.10, *z* = −5.75, *p* < 0.001, OR = 0.56). Moreover, a difference between the Nap and Wake groups across the T0 to T1 sessions was found, indicating that the change in arousal from T0 to T1 is greater in the Nap group compared to the Wake group (i.e., the Nap group showed a stronger reduction in arousal ratings for neutral images from T0 to T1) (log‐odds = 0.17, SE = 0.06, *z* = 2.68, *p* = 0.007, OR = 1.18). Additionally, a difference between the Nap and Wake groups across the T1 to T2 sessions was found, revealing that the two groups behave differently from T1 to T2. Specifically, the Wake group showed a reduction in arousal ratings, while the Nap group exhibited an increase in arousal ratings (log‐odds = −0.21, SE = 0.06, *z* = −3.26, *p* = 0.001, OR = 0.81), as synthesized in Figure 4. Full model results can be found in Table S6.

**FIGURE 4:**
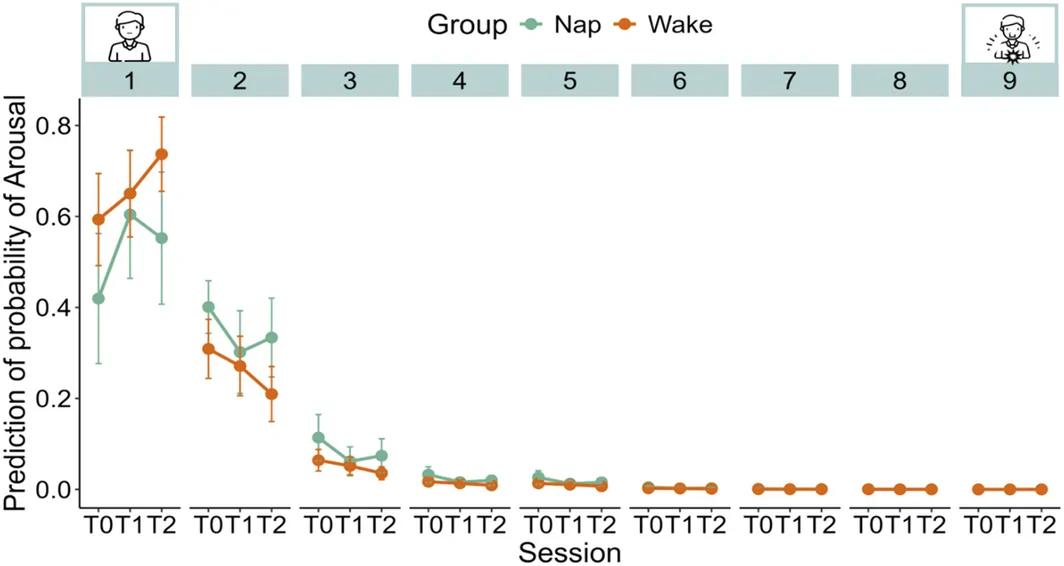
Predicted probability of Arousal for neutral images. Comparison between Nap and Wake groups across the three sessions (T0, T1, T2). Each dot represents the estimated mean probability for each ordinal level. Error bars represent the standard error estimated by the model. Numbers from 1 to 9 above the plot indicate ordinal responses on the Self‐Assessment Manikin, with 1 corresponding to low arousal levels and 9 to high arousal levels.

#### 3.4.2 Valence

The cumulative link model conducted on Valence to assess changes in subjective valence for negative images, with Group and Session as fixed effects, and subjects as random intercept, revealed a difference between T1 and T2, with lower scores at T1 compared to T2 (i.e., negative images were evaluated as less pleasant at T1 than at T2) (log‐odds = 0.25, SE = 0.09, *z* = 2.66, *p* = 0.008, OR = 1.28). Moreover, a difference between the REM and No‐REM groups across the T1 to T2 sessions was found, indicating that the decrease for negative images was higher in the REM group than in the No‐REM group (i.e., negative images were evaluated as less pleasant at T1 than at T2 in the REM group) (log‐odds = 0.50, SE = 0.13, *z* = 3.97, *p* < 0.001, OR = 1.64), as shown in Figure 5a. Additionally, a difference between the Nap and Wake groups across the T1 to T2 sessions was found, indicating that the Nap group showed a greater decrease in ratings for negative images compared to the Wake group (log‐odds = −0.16, SE = 0.06, *z* = −2.70, *p* = 0.007, OR = 0.86), as shown in Figure 5b. Full model results can be found in Table S8.

**FIGURE 5:**
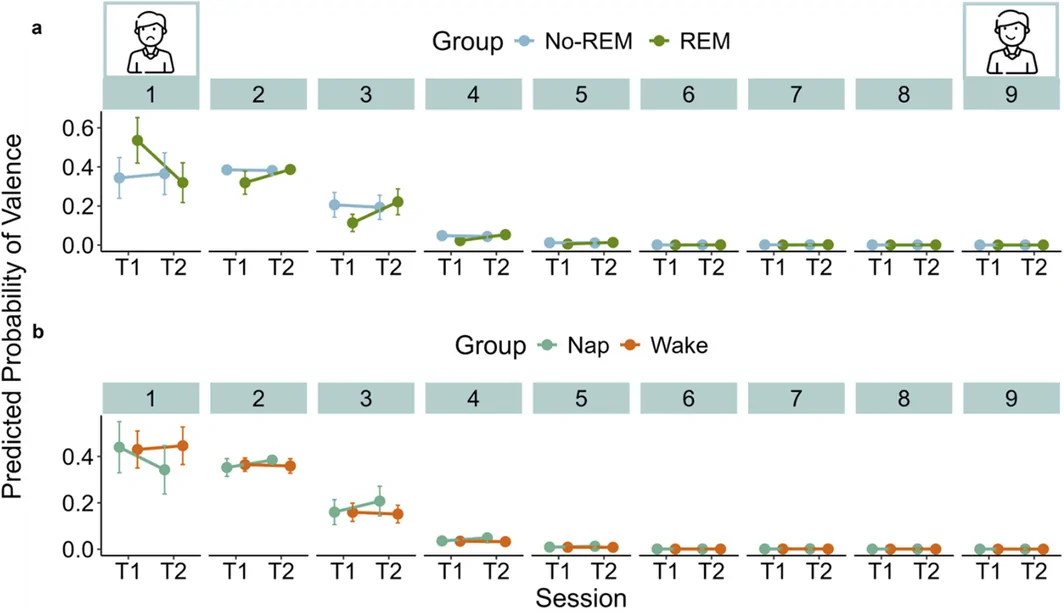
Predicted probability of Valence for negative images across T1 and T2. (a) Comparison between the No‐REM and REM groups. (b) Comparison between the Nap and Wake groups. Each dot represents the mean probability estimated by the model for each ordinal level. Error bars represent the standard error estimated by the model. Numbers from 1 to 9 above the plot indicate ordinal responses on the Self‐Assessment Manikin, with 1 corresponding to low valence (very negative image) and 9 to high valence (very positive image).

The cumulative link model conducted on Valence to assess changes in valence for neutral images, with Group and Session as fixed effects, and subjects as random intercept, revealed a difference between T0 and T1, with lower scores at T1 compared to T0 (i.e., neutral images were evaluated as less pleasant at T1 compared to T0) (log‐odds = −0.27, SE = 0.09, *z* = −2.97, *p* = 0.003, OR = 0.76). Moreover, a difference between T1 and T2 was observed, with lower ratings at T2 compared to T1 (i.e., neutral images were evaluated as less pleasant at T2 compared to T1) (log‐odds = −0.27, SE = 0.09, *z* = −2.86, *p* = 0.004, OR = 0.77). Full model results can be found in Table S9.

#### 3.4.3 Skin Conductance Response

The generalized linear mixed model conducted to assess changes in SCR for negative images, with Group and Session as fixed effects, and subjects as random intercept, revealed a difference between T1 and T0, with greater SCR at T0 compared to T1 (*β* = −0.41, SE = 0.11, *t* = −3.62, *p* < 0.001, β* = −0.36). Full model results can be found in Table S11.

The generalized linear mixed model conducted to assess changes in SCR to neutral images, with Group and Session as fixed effects, and subjects as random intercept, revealed a difference between T1 and T0, with greater SCR at T0 compared to T1 (*β* = −0.32, SE = 0.11, *t* = −2.98, *p* = 0.003, β* = −0.32). Full model results can be found in Table S12.

#### 3.4.4 Phasic Cardiac Response (∆PCR)

The linear mixed model conducted on ∆PCR to assess cardiac response to negative images, with Group and Session as fixed effects, subjects as random intercept, and Time of image presentation nested within Session, revealed a difference between the REM and No‐REM groups across T0 and T1. The REM group showed an increase in cardiac deceleration from T0 to T1, resulting in more negative ∆PCR values at T1, while the No‐REM group exhibited the opposite pattern, decreasing its cardiac deceleration from T0 to T1, resulting in less negative ∆PCR values at T1 (*β* = −0.57, SE = 0.17, *t* = −3.33, *p* < 0.001, β* = −0.25) (see Figure 6). Full model results can be found in Table S14.

**FIGURE 6:**
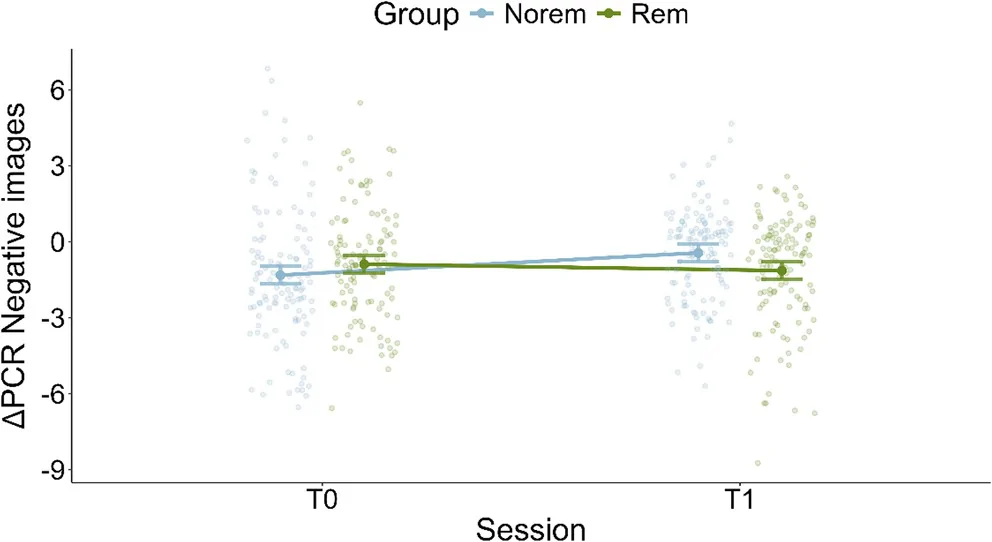
∆PCR for negative images. Change in Phasic Cardiac Response (∆PCR) in No‐REM and REM groups across sessions (T0, T1). Bigger dots represent the mean estimated by the model. Smaller dots represent individual subjects' values. Error bars represent the standard error estimated by the model.

The linear mixed model conducted on ∆PCR to assess cardiac response changes to neutral images, with Group and Session as fixed effects, subjects as random intercept, and Time of image presentation nested within Session, showed a difference between the Wake and the Nap group. The Wake group showed lower cardiac deceleration (i.e., more positive ∆PCR values) than the Nap group (*β* = 0.15, SE = 0.07, *t* = 2.04, *p* = 0.045, β* = 0.08). Additionally, the results indicated a difference between T1 and T0 with lower cardiac deceleration at T1 compared to T0 (*β* = 0.53, SE = 0.22, *t* = 2.39, *p* = 0.029, β* = 0.27). Full model results can be found in Table S15.

## 4 Discussion

Our study aimed to assess the role of sleep, specifically an afternoon nap, on emotional processing at both behavioral and physiological levels. We examined the effects of sleep separately on emotional memory and emotional reactivity using two distinct picture presentation tasks. Participants were exposed to the tasks before and after a manipulation condition, in which half of the participants took a nap while the other half remained awake. Emotional processing was further assessed in the long‐term using the same tasks 2 days later. Beyond investigating the general effects of sleep, the employment of a nap paradigm allowed us to explore the role of different sleep stages (i.e., NREM and REM) in emotional processing through the use of the nap duration without directly manipulating sleep architecture. However, the two groups differed in TST, WASO, and N3%, while participants who reached REM sleep showed substantial variability in the amount of time spent in this stage. These structural differences may be relevant to emotional processing when considering the specific contribution of individual sleep stages. When examining the effects of TST, N3%, and REM% on emotional memory, none of these parameters were associated with memory performance immediately after the nap. Conversely, when considering the impact of TST on subjective arousal, this effect was attenuated during the test immediately following the nap. This finding appears to reflect a broader modulatory role of sleep rather than an effect attributable to a specific sleep stage. We also found a modulatory effect of N3% on PCR, with PCR becoming more attenuated as time spent in N3 increased. Notably, the REM group also spent more time in N3 than the No‐REM group. Considering these findings, in light of Walker's model, which proposes that REM sleep contributes to the attenuation of emotional reactivity, this pattern may suggest that the modulation of emotional reactivity depends not only on the presence of REM sleep, but also on the broader organization of sleep architecture. Moreover, we found no association between time spent in REM and measures of emotional reactivity. Taken together, these findings may suggest that, while even relatively brief periods of REM sleep may contribute to emotional modulation, the amount of N3 sleep may also play a role in shaping physiological emotional reactivity.

Concerning memory performance, we observed an effect of sleep, with the Nap group showing less memory decline than the Wake group immediately after the manipulation condition (T1). This effect was evident regardless of image type, as both neutral and negative images benefited from sleep. This finding is particularly debated in the literature: some studies report no preferential consolidation of emotional memories during sleep (Bolinger et al. 2018; Cellini et al. 2016; Cunningham, Crowell, et al. 2014), while others demonstrate a greater preservation of emotional memories following sleep (Chambers and Payne 2014; Prehn‐Kristensen et al. 2017; Wagner et al. 2006). According to a meta‐analysis conducted by Lipinska et al. (2019), these inconsistencies may result from methodological differences, such as the type of memory test used, with free recall tests showing the strongest preferential effect of sleep on emotional memories. Since our memory test was based on recognition, it is possible that this effect was more difficult to detect.

However, the effect of sleep on memory varied by image type in the long term (T2), as the difference in memory performance between the Nap and Wake groups persisted only for the negative images, not for the neutral ones. This finding may indicate that the preferential role of sleep in the consolidation of emotional memories is detectable in the long term, and that shorter intervals between an emotional event and the onset of sleep may benefit this process, as indicated by the long‐term effect observed on the memory performance for the negative images. Supporting this idea, previous studies have shown that the timing of sleep following the encoding of traumatic memories influences emotional processing, leading to reduced emotional reactivity and fewer intrusive traumatic memories in participants who slept shortly after exposure to the emotional events (Werner et al. 2021; Wilhelm et al. 2021).

In addition, no differences were detected between the No‐REM and REM groups, suggesting that REM sleep may not have played a role in emotional memory consolidation. This result contrasts with several studies reporting a positive association between REM‐rich sleep and emotional memory consolidation (Gilson et al. 2015; Groch et al. 2013; Wagner et al. 2001). However, these findings have recently been found to be inconsistent, possibly due to different methodologies employed to isolate sleep stages, such as through nap paradigms, split‐night designs, or stage deprivation methods (Schäfer et al. 2020). Furthermore, the proposed beneficial role of REM sleep in emotional memory consolidation has been further questioned by a series of studies employing targeted memory reactivation (TMR) during both NREM and REM sleep (Cairney et al. 2014; Lehmann et al. 2016; Yuksel et al. 2025). These studies reported positive effects of TMR only when applied during NREM sleep, with Yuksel et al. (2025) even observing increased forgetting when TMR was applied during REM sleep. Overall, the absence of group differences in our findings is consistent with the growing body of evidence suggesting a preferential role of NREM over REM sleep in the consolidation of emotional memories. However, unlike previous studies that have found an association between SWS and memory for negative stimuli (Alger et al. 2018; Payne et al. 2015), in our study, time spent in N3 was not significantly associated with memory performance. This finding may suggest that emotional processing requires a sequential progression through sleep stages, and that NREM and REM sleep may act synergistically on this process.

Emotional reactivity was assessed using both subjective image evaluations and physiological responses. Regarding the subjective ratings, we observed an effect of sleep on arousal in response to negative images, with the Nap group showing a decrease in arousal ratings after the manipulation condition (T1) compared to the Wake group. This effect was also evident in the REM group, which exhibited a larger decrease in arousal ratings at T1 compared to the No‐REM group. An effect of sleep on subjective arousal was also observed for neutral images, with the Nap group again showing a greater immediate reduction in arousal after sleep, followed by a rebound 2 days later. This persistence may be partially explained by the nature of the neutral stimuli, which were sport‐related and may have elicited activation in some participants.

In contrast, no effect of sleep was observed for subjective ratings of valence immediately after the manipulation. However, in the long term, participants in the Nap group rated negative images as less negative than those in the Wake group. A similar pattern was observed in the REM group compared to the No‐REM group. This delayed effect on valence ratings, which did not occur for arousal ratings, was also observed in a study where participants, who slept immediately after being presented with negative images, showed initial preservation of valence ratings, followed by a decrease after 60 h (Zeng et al. 2021). This long‐term effect on subjective valence of sleep, along with the long‐term effect on emotional memory, may indicate that the interval between an emotional event and subsequent sleep could play a role in emotional processing, particularly for emotional responses that involve cognitive control.

Taken together, these findings suggest that REM sleep, or more precisely the succession of the two sleep stages (i.e., NREM and REM), might reduce subjective emotional reactivity, although in different modalities, with the effect on arousal appearing to be immediate, whereas the effect on valence emerges gradually over time, as also reported by recent studies (Bolinger et al. 2019; Zeng et al. 2021). These results support the SFSR hypothesis, according to which sleep attenuates the emotional tone of an event (Walker and Van Der Helm 2009). Nevertheless, despite most studies assessed subjective reactivity by asking participants to rate arousal and valence (Davidson and Pace‐Schott 2021), findings in the literature remain highly variable. For example, some studies have reported an increased or preserved emotional reactivity after sleep (Jones and Spencer 2019; Wagner 2002; Wilhelm et al. 2021), while others observed a decreased reactivity (Bolinger et al. 2019; Cunningham, Chambers, and Payne 2014; van der Helm et al. 2011) or a specific role of REM sleep revealed through the TMR paradigm (Hutchison et al. 2021). Still, other studies have found no effect at all (Cox et al. 2018; Harrington et al. 2018). In addition, even within the same study, results for valence and arousal do not always align (Davidson and Pace‐Schott 2021), as partially observed in our study.

Although the majority of studies investigating emotional reactivity have relied on subjective measures, which are more influenced by cognitive processes, some studies have employed physiological responses. Notably, physiological responses are highly automatic and less susceptible to cognitive biases (Bolinger et al. 2018). In our study, we used two of the most commonly used physiological measures in emotional reactivity research: phasic cardiac response (PCR) and skin conductance response (SCR). The most common PCR is the HR deceleration (HRD), an orienting response mediated by the parasympathetic nervous system that may reflect the valence of a stimulus, as it is accentuated by negative stimuli (Graham and Clifton 1966). SCR is mediated by the sympathetic nervous system and is considered an index of physiological arousal (Cox et al. 2023).

Results on PCR indicate an increase in emotional reactivity in the REM group after the nap compared to the No‐REM group, with the REM group showing greater HRD. This finding is supported by some studies reporting an increased HRD in participants who slept (Bolinger et al. 2018, 2019; Pace‐Schott et al. 2011), but contrasts with others that observed a decrease in emotional reactivity, as reflected by reduced HRD following sleep (Cunningham, Crowell, et al. 2014; Jones and Spencer 2019). It is important to note that we found a specific effect of sleep stages on PCR, whereas previous research primarily examined general sleep effects and often failed to find correlations between sleep parameters, such as REM duration, and cardiac response (Davidson and Pace‐Schott 2021).

Unlike other measures, SCR did not differ between groups, which is consistent with previous studies reporting no significant effects of sleep on SCR (Ashton et al. 2019; Lipinska and Thomas 2019). However, as with other emotional reactivity measures, findings in the literature are highly variable. Some studies have reported a decrease in SCR after sleep (Cunningham, Crowell, et al. 2014), but only when participants did not enter REM sleep (Pace‐Schott et al. 2011). Conversely, other research has found a preservation of SCR after a period of sleep compared to a period of wakefulness (Jones and Spencer 2019). One potential explanation for the inconsistency in SCR findings could be that these studies did not control for its diurnal variability. Hot et al. (2005) demonstrated that SCR fluctuates across the day, reaching its maximum peak in the afternoon and its minimum in the morning. This aspect was not accounted for in many studies comparing participants at different times of the day (i.e., after a night of sleep versus after a day of wakefulness).

Our results should be interpreted in light of some limitations. Several of these relate to the tasks employed. First, we used pictures in both tasks, which, although highly negative, cannot be considered either ecological or realistic representations of emotional events. Future research should employ more ecologically valid paradigms, such as creating emotional events in controlled settings, as previously accomplished by Wassing et al. (2019). Second, the emotional reactivity task lacked novel stimuli as control items. Including such stimuli would have allowed us to determine whether the observed sleep effect was specific to the processing of already acquired information, or instead reflected a broader influence on emotional reactivity (Davidson and Pace‐Schott 2021). Third, in the memory task, a recall test could have been used instead of a recognition test, as recall is more sensitive to the preferential effect of sleep on emotional memory (Lipinska et al. 2019). Another weakness of our study concerns the paradigm we employed to investigate the role of different sleep stages in emotional processing. The nap paradigm allowed us to test all groups at the same time of day, thereby minimizing circadian influences, which are known to affect EDA and HR (Hot et al. 2005; Vandewalle et al. 2007). However, although this approach enabled us to observe the effects of NREM sleep or the succession of NREM and REM sleep stages, it did not allow us to clarify whether the effect was driven by REM sleep alone or by the interaction between stages. Moreover, this paradigm allowed us to observe the effects of a single sleep cycle on emotional processing, reducing a process that may naturally need many sleep cycles or even several nights to be accomplished. Although more natural, the choice of this paradigm introduced architectural differences between the two groups in terms of total sleep time and sleep depth, making it difficult to discriminate whether the results are a mere effect of an overall lower sleep quality of the No‐REM group or an effect of sleep stages succession. It should be noted that differences in sleep architecture are common in studies employing this paradigm to discriminate between REM and No‐REM during a nap, with studies typically reporting greater TST in the REM group (Cellini et al. 2016) and greater WASO and time spent in N1 in the No‐REM group (Werner et al. 2021). However, the differences observed in the present study appear more accentuated than those reported in previous studies, which may partly reflect the absence of adaptation sessions and the lack of instructions to advance wake time to increase sleep pressure, factors that future studies should consider to obtain more balanced groups. From additional analyses conducted to assess the effect of total sleep time and percentage of REM sleep on the variables examined, we did not find an effect of REM percentage, while TST was associated with subjective arousal to negative images. This pattern suggests that sleep duration may contribute to some of the observed effects, and that future studies should include designs that better isolate REM sleep while controlling for overall sleep quantity. We also found an effect of N3 sleep stage, indicating a change from T0 to T1 of PCR into more positive values when the time spent in N3 was higher. Interestingly, this pattern differed from the effect observed between REM and No‐REM participants, where the REM group showed increased cardiac deceleration at T1. These findings suggest that N3 sleep may contribute to the modulation of cardiac responses following sleep, but it is unlikely to entirely account for the effects observed in the participants entering REM sleep.

It should be noted that, due to the difficulty of identifying participants capable of falling asleep in the afternoon within the unfamiliar environment of a sleep laboratory, we recruited individuals with a habitual propensity for napping to maximize the probability of successful sleep onset. Nevertheless, this represents a significant methodological limitation; although the groups showed significant differences only regarding daytime sleepiness, the study's results must be interpreted considering this a priori difference between the two groups. Lastly, we did not collect any measure of sleep quality and quantity in the days preceding and during the experiment, although participants were instructed to maintain their usual sleep habits.

To summarize, we conducted a between‐subjects study employing a nap paradigm, obtaining one of the largest sample sizes in this research field (Lipinska et al. 2019). In agreement with the SFSR hypothesis and theories on the memory consolidation role of sleep (Diekelmann and Born 2010), we observed better memory performance after sleep compared to wakefulness. However, we did not find any effect of REM sleep on emotional memory. Regarding emotional reactivity, our results were inconsistent across and within subjective and physiological measures. Subjective ratings were in line with the SFSR hypothesis, showing a decrease in participants who also experienced REM sleep. However, this effect was immediate for arousal ratings, whereas valence ratings were initially preserved, with a reduction observed 2 days later. Physiological measures, on the other hand, contradicted the SRSF hypothesis. SCR showed no effect of sleep, whereas PCR supported the ESC hypothesis (Baran et al. 2012), with an enhancement of the emotional reactivity after REM sleep.

Divergent results between physiological and subjective measures have already been reported in previous studies (Bolinger et al. 2018; Jones and Spencer 2019; Reinhold et al. 2025), suggesting that physiological responses and cognitive evaluations of emotional events may underlie different mechanisms, and that emotional reactivity must be investigated by distinguishing them. In this case, sleep, and in particular REM sleep, or the succession of NREM and REM sleep stages, seems to decrease the emotional reactivity of responses subjected to cognitive control, while enhancing reactivity in more automatic physiological responses, although it cannot be excluded that these differences may partly reflect broader differences in sleep quality between groups.

Taken together, these findings provide a broader perspective on the contribution of afternoon naps to emotional processing and reactivity, suggesting that even relatively brief sleep episodes may modulate these processes through multiple aspects of sleep architecture, including REM sleep, total sleep duration, and deep sleep. Overall, the results underscore the complexity of sleep‐related emotional processing and highlight the need for rigorous experimental paradigms capable of disentangling the specific contributions of different sleep stages.

As a final note, the contradictory findings in studies measuring emotional reactivity may stem from substantial variability in stimuli, experimental protocols (e.g., different methods of sleep manipulation), outcome variables, data analyses, and different effects of sleep stages (Davidson and Pace‐Schott 2021). Therefore, future research should employ standardized protocols, consistent variables, and well‐powered designs. Additionally, studies conducted in more ecological environments are needed to ensure the applicability of findings to real‐life contexts.

## Author Contributions

**Costanza Colombo:** investigation, data curation, formal analysis, writing – review and editing, software, visualization, writing – original draft. **Angie Baldassarri:** investigation, writing – original draft, visualization, writing – review and editing, software, formal analysis, data curation. **Umberto Granziol:** formal analysis, writing – review and editing. **Jessica Ongaro:** writing – review and editing, investigation. **Lorenzo Viselli:** software, methodology, writing – review and editing. **Michele Ferrara:** conceptualization, writing – review and editing. **Aurora D'Atri:** funding acquisition, conceptualization, writing – review and editing. **Nicola Cellini:** conceptualization, methodology, project administration, resources, supervision, validation, writing – review and editing, funding acquisition.

## Funding

This study was supported by funding from the European Union—NextGenerationEU—National Recovery and Resilience Plan (NRRP)—MISSION 4 COMPONENT 2, INVESTMENT N. 1.1, CALL PRIN 2022 D.D. 104 02‐02‐2022, Project Title: ‘How Emotional Reactivity and Memory Evolve Over Sleep: Psychophysiological Description via Exogenous manipulation of Sleep features (HERMES‐DEUS)’, awarded to ADA and N.C. (CUP N. E53D23008840006) through the Ministry of University and Research (MUR).

## Ethics Statement

All participants provided informed consent. The study protocol was approved by the local Ethics Committee (Comitato Etico della Ricerca Psicologica, area 17). Code: E3E80B20F9BC7D87B30E7104552D89DE.

## Conflicts of Interest

This is not an industry‐supported study. None of the authors has potential financial or non‐financial conflicts of interest to be disclosed. All authors have seen and approved the manuscript.

## Supporting information


**Table S1:** Full model results of d‐prime analysis at T0.
**Table S2:** Full model results of d‐prime analysis on negative images.
**Table S3:** Full model results of d‐prime analysis on neutral images.
**Table S4:** Full model results on Arousal at T0.
**Figure S1:** Predicted probability of Arousal at T0. (a) Comparison between negative and neutral images for No‐REM and REM groups. (b) Comparison between negative and neutral images for Nap and Wake groups.
Each dot represents the mean probability estimated by the model for each ordinal level. Error bars represent the standard error estimated by the model. Numbers from 1 to 9 above the plot indicate ordinal responses on the Self‐Assessment Manikin, with 1 corresponding to low arousal levels and 9 to high arousal levels.
**Table S5:** Full model results on Arousal on negative images.
**Table S6:** Full model results on Arousal on neutral images.
**Figure S2:** Predicted probability of Valence at T0. (a) Comparison between negative and neutral images for No‐REM and REM groups (b). Comparison between negative and neutral images for the Nap and Wake groups. Each dot represents the mean probability estimated by the model for each ordinal level. Error bars represent the standard error estimated by the model. Numbers from 1 to 9 above the plot indicate ordinal responses on the Self‐Assessment Manikin, with 1 corresponding to low valence (very negative image) and 9 to high valence (very positive image).
**Table S7:** Full model results on Valence at T0.
**Table S8:** Full model results on Valence on negative images.
**Table S9:** Full model results on Valence on neutral images.
**Table S10:** Full model results on SCR at T0 with and without outliers.
**Table S11:** Full model results on SCR for negative images.
**Table S12:** Full model results on SCR for neutral images.
**Table S13:** Full model results on ΔPCR analysis at T0.
**Table S14:** Full model results on ΔPCR on negative images.
**Table S15:** Full model results on ΔPCR on neutral images.
**Table S16:** Arousal and Valence ratings for each image.
**Table S17:** Mean Arousal and Valence ratings for each image set.
**Table S18:** Means valence and arousal ratings for negative and neutral images of the emotional reactivity task and their ID.
**Table S19:** Mean valence and arousal ratings for negative and neutral images of the emotional memory task and their ID.
**Table S20:** Contingency table of picture‐set assignment across groups.

## Data Availability

All datasets and code underlying the results are publicly available in the Open Science Framework repository at https://osf.io/5htjd/.

## References

[psyp70414-bib-0001] Adan, A. , and H. Almirall . 1991. “Horne & Östberg Morningness‐Eveningness Questionnaire: A Reduced Scale.” Personality and Individual Differences 12, no. 3: 241–253. 10.1016/0191-8869(91)90110-W.

[psyp70414-bib-0002] Agresti, A. 2010. Analysis of Ordinal Categorical Data. 1st ed. Wiley. 10.1002/9780470594001.

[psyp70414-bib-0003] Alger, S. E. , E. A. Kensinger , and J. D. Payne . 2018. “Preferential Consolidation of Emotionally Salient Information During a Nap Is Preserved in Middle Age.” Neurobiology of Aging 68: 34–47. 10.1016/j.neurobiolaging.2018.03.030. PMC5993606 29704647

[psyp70414-bib-0004] Alger, S. E. , and J. D. Payne . 2016. “The Differential Effects of Emotional Salience on Direct Associative and Relational Memory During a Nap.” Cognitive, Affective, & Behavioral Neuroscience 16, no. 6: 1150–1163. 10.3758/s13415-016-0460-1. 27670288

[psyp70414-bib-0005] Ashton, J. E. , M. O. Harrington , A. Á. V. Guttesen , A. K. Smith , and S. A. Cairney . 2019. “Sleep Preserves Physiological Arousal in Emotional Memory.” Scientific Reports 9, no. 1: 5966. 10.1038/s41598-019-42478-2. PMC6461689 30979941

[psyp70414-bib-0006] Baran, B. , E. F. Pace‐Schott , C. Ericson , and R. M. C. Spencer . 2012. “Processing of Emotional Reactivity and Emotional Memory Over Sleep.” Journal of Neuroscience 32, no. 3: 1035–1042. 10.1523/JNEUROSCI.2532-11.2012. PMC3548452 22262901

[psyp70414-bib-0007] Bastien, C. 2001. “Validation of the Insomnia Severity Index as an Outcome Measure for Insomnia Research.” Sleep Medicine 2, no. 4: 297–307. 10.1016/S1389-9457(00)00065-4. 11438246

[psyp70414-bib-0008] Bates, D. , M. Mächler , B. Bolker , and S. Walker . 2015. “Fitting Linear Mixed‐Effects Models Using lme4.” Journal of Statistical Software 67, no. 1: 1–48. 10.18637/jss.v067.i01.

[psyp70414-bib-0009] Benedek, M. , and C. Kaernbach . 2010. “A Continuous Measure of Phasic Electrodermal Activity.” Journal of Neuroscience Methods 190, no. 1: 80–91. 10.1016/j.jneumeth.2010.04.028. PMC2892750 20451556

[psyp70414-bib-0010] Bolinger, E. , J. Born , and K. Zinke . 2018. “Sleep Divergently Affects Cognitive and Automatic Emotional Response in Children.” Neuropsychologia 117: 84–91. 10.1016/j.neuropsychologia.2018.05.015. 29782873

[psyp70414-bib-0011] Bolinger, E. , T. J. Cunningham , J. D. Payne , et al. 2019. “Sleep's Benefits to Emotional Processing Emerge in the Long Term.” Cortex 120: 457–470. 10.1016/j.cortex.2019.07.008. 31476555

[psyp70414-bib-0012] Bottesi, G. , M. Ghisi , G. Altoè , E. Conforti , G. Melli , and C. Sica . 2015. “The Italian Version of the Depression Anxiety Stress Scales‐21: Factor Structure and Psychometric Properties on Community and Clinical Samples.” Comprehensive Psychiatry 60: 170–181. 10.1016/j.comppsych.2015.04.005. 25933937

[psyp70414-bib-0013] Bradley, M. M. , and P. J. Lang . 1994. “Measuring Emotion: The Self‐Assessment Manikin and the Semantic Differential.” Journal of Behavior Therapy and Experimental Psychiatry 25, no. 1: 49–59. 10.1016/0005-7916(94)90063-9. 7962581

[psyp70414-bib-0014] Bradley, M. M. , and P. J. Lang . 2007. “The International Affective Picture System (IAPS) in the Study of Emotion and Attention.” In Handbook of Emotion Elicitation and Assessment, edited by J. A. Coan and J. J. B. Allen . Oxford University Press. 10.1093/oso/9780195169157.003.0003.

[psyp70414-bib-0015] Brain Products GmbH . 2021. BrainVision Recorder. Brain Products GmbH.

[psyp70414-bib-0016] Buysse, D. J. , C. F. Reynolds , T. H. Monk , S. R. Berman , and D. J. Kupfer . 1989. “The Pittsburgh Sleep Quality Index: A New Instrument for Psychiatric Practice and Research.” Psychiatry Research 28, no. 2: 193–213. 10.1016/0165-1781(89)90047-4. 2748771

[psyp70414-bib-0017] Cabrera, Y. , K. J. Koymans , G. R. Poe , H. W. Kessels , E. J. W. Van Someren , and R. Wassing . 2024. “Overnight Neuronal Plasticity and Adaptation to Emotional Distress.” Nature Reviews Neuroscience 25, no. 4: 253–271. 10.1038/s41583-024-00799-w. 38443627

[psyp70414-bib-0018] Cairney, S. A. , S. J. Durrant , J. Hulleman , and P. A. Lewis . 2014. “Targeted Memory Reactivation During Slow Wave Sleep Facilitates Emotional Memory Consolidation.” Sleep 37, no. 4: 701–707. 10.5665/sleep.3572. PMC3954173 24688163

[psyp70414-bib-0019] Castronovo, V. , A. Galbiati , S. Marelli , et al. 2016. “Validation Study of the Italian Version of the Insomnia Severity Index (ISI).” Neurological Sciences 37, no. 9: 1517–1524. 10.1007/s10072-016-2620-z. 27234459

[psyp70414-bib-0020] Cellini, N. , J. Torre , L. Stegagno , and M. Sarlo . 2016. “Sleep Before and After Learning Promotes the Consolidation of Both Neutral and Emotional Information Regardless of REM Presence.” Neurobiology of Learning and Memory 133: 136–144. 10.1016/j.nlm.2016.06.015. 27321589

[psyp70414-bib-0021] Chambers, A. M. , and J. D. Payne . 2014. “Laugh Yourself to Sleep: Memory Consolidation for Humorous Information.” Experimental Brain Research 232, no. 5: 1415–1427. 10.1007/s00221-013-3779-7. 24337230

[psyp70414-bib-0022] Christensen, R. 2023. ordinal—Regression Models for Ordinal Data. https://CRAN.R‐project.org/package=ordinal.

[psyp70414-bib-0023] Cox, O. D. , A. Munjal , W. V. McCall , B. J. Miller , C. Baeken , and P. B. Rosenquist . 2023. “A Review of Clinical Studies of Electrodermal Activity and Transcranial Magnetic Stimulation.” Psychiatry Research 329: 115535. 10.1016/j.psychres.2023.115535. 37839318

[psyp70414-bib-0024] Cox, R. , M. L. V. Van Bronkhorst , M. Bayda , et al. 2018. “Sleep Selectively Stabilizes Contextual Aspects of Negative Memories.” Scientific Reports 8, no. 1: 17861. 10.1038/s41598-018-35999-9. PMC6294767 30552343

[psyp70414-bib-0025] Cunningham, T. J. , A. M. Chambers , and J. D. Payne . 2014. “Prospection and Emotional Memory: How Expectation Affects Emotional Memory Formation Following Sleep and Wake.” Frontiers in Psychology 5: 862. 10.3389/fpsyg.2014.00862. PMC4120689 25136328

[psyp70414-bib-0026] Cunningham, T. J. , C. R. Crowell , S. E. Alger , et al. 2014. “Psychophysiological Arousal at Encoding Leads to Reduced Reactivity but Enhanced Emotional Memory Following Sleep.” Neurobiology of Learning and Memory 114: 155–164. 10.1016/j.nlm.2014.06.002. 24952130

[psyp70414-bib-0027] Cunningham, T. J. , and J. D. Payne . 2017. “Emotional Memory Consolidation During Sleep.” In Cognitive Neuroscience of Memory Consolidation, edited by N. Axmacher and B. Rasch , 133–159. Springer International Publishing. 10.1007/978-3-319-45066-7_9.

[psyp70414-bib-0028] Curcio, G. , D. Tempesta , S. Scarlata , et al. 2013. “Validity of the Italian Version of the Pittsburgh Sleep Quality Index (PSQI).” Neurological Sciences 34, no. 4: 511–519. 10.1007/s10072-012-1085-y. 22526760

[psyp70414-bib-0029] Davidson, P. , P. Jönsson , I. Carlsson , and E. Pace‐Schott . 2021. “Does Sleep Selectively Strengthen Certain Memories Over Others Based on Emotion and Perceived Future Relevance?” Nature and Science of Sleep 13: 1257–1306. 10.2147/NSS.S286701. PMC8318217 34335065

[psyp70414-bib-0030] Davidson, P. , and E. Pace‐Schott . 2021. “Go to Bed and You MIGHT Feel Better in the Morning—The Effect of Sleep on Affective Tone and Intrusiveness of Emotional Memories.” Current Sleep Medicine Reports 7, no. 2: 31–46. 10.1007/s40675-020-00200-z.

[psyp70414-bib-0031] Diekelmann, S. , and J. Born . 2010. “The Memory Function of Sleep.” Nature Reviews Neuroscience 11, no. 2: 114–126. 10.1038/nrn2762. 20046194

[psyp70414-bib-0032] Gambarota, F. , and G. Altoè . 2024. “Ordinal Regression Models Made Easy: A Tutorial on Parameter Interpretation, Data Simulation and Power Analysis.” International Journal of Psychology 59, no. 6: 1263–1292. 10.1002/ijop.13243. 39354666

[psyp70414-bib-0033] Genzel, L. , V. I. Spoormaker , B. N. Konrad , and M. Dresler . 2015. “The Role of Rapid Eye Movement Sleep for Amygdala‐Related Memory Processing.” Neurobiology of Learning and Memory 122: 110–121. 10.1016/j.nlm.2015.01.008. 25638277

[psyp70414-bib-0034] Gilson, M. , G. Deliens , R. Leproult , et al. 2015. “REM‐Enriched Naps Are Associated With Memory Consolidation for Sad Stories and Enhance Mood‐Related Reactivity.” Brain Sciences 6, no. 1: 1. 10.3390/brainsci6010001. PMC4810171 26729175

[psyp70414-bib-0035] Giuditta, A. 2014. “Sleep Memory Processing: The Sequential Hypothesis.” Frontiers in Systems Neuroscience 8: 219. 10.3389/fnsys.2014.00219. PMC4267175 25565985

[psyp70414-bib-0036] Graham, F. K. , and R. K. Clifton . 1966. “Heart‐Rate Change as a Component of the Orienting Response.” Psychological Bulletin 65, no. 5: 305–320. 10.1037/h0023258. 5325894

[psyp70414-bib-0037] Groch, S. , I. Wilhelm , S. Diekelmann , and J. Born . 2013. “The Role of REM Sleep in the Processing of Emotional Memories: Evidence From Behavior and Event‐Related Potentials.” Neurobiology of Learning and Memory 99: 1–9. 10.1016/j.nlm.2012.10.006. 23123802

[psyp70414-bib-0038] Groch, S. , K. Zinke , I. Wilhelm , and J. Born . 2015. “Dissociating the Contributions of Slow‐Wave Sleep and Rapid Eye Movement Sleep to Emotional Item and Source Memory.” Neurobiology of Learning and Memory 122: 122–130. 10.1016/j.nlm.2014.08.013. 25180933

[psyp70414-bib-0039] Gujar, N. , S. A. McDonald , M. Nishida , and M. P. Walker . 2011. “A Role for REM Sleep in Recalibrating the Sensitivity of the Human Brain to Specific Emotions.” Cerebral Cortex 21, no. 1: 115–123. 10.1093/cercor/bhq064. PMC3000566 20421251

[psyp70414-bib-0040] Harrington, M. O. , K. M. Nedberge , and S. J. Durrant . 2018. “The Effect of Sleep Deprivation on Emotional Memory Consolidation in Participants Reporting Depressive Symptoms.” Neurobiology of Learning and Memory 152: 10–19. 10.1016/j.nlm.2018.04.013. 29709569

[psyp70414-bib-0041] Hautus, M. J. , N. A. Macmillan , and C. D. Creelman . 2021. Detection Theory: A User's Guide. 3rd ed. Routledge. 10.4324/9781003203636.

[psyp70414-bib-0042] Henry, J. D. , and J. R. Crawford . 2005. “The Short‐Form Version of the Depression Anxiety Stress Scales (DASS‐21): Construct Validity and Normative Data in a Large Non‐Clinical Sample.” British Journal of Clinical Psychology 44, no. 2: 227–239. 10.1348/014466505X29657. 16004657

[psyp70414-bib-0043] Hot, P. , P. Leconte , and H. Sequeira . 2005. “Diurnal Autonomic Variations and Emotional Reactivity.” Biological Psychology 69, no. 3: 261–270. 10.1016/j.biopsycho.2004.08.005. 15925029

[psyp70414-bib-0044] Hu, P. , M. Stylos‐Allan , and M. P. Walker . 2006. “Sleep Facilitates Consolidation of Emotional Declarative Memory.” Psychological Science 17, no. 10: 891–898. 10.1111/j.1467-9280.2006.01799.x. 17100790

[psyp70414-bib-0045] Hutchison, I. C. , S. Pezzoli , M.‐E. Tsimpanouli , et al. 2021. “Targeted Memory Reactivation in REM but Not SWS Selectively Reduces Arousal Responses.” Communications Biology 4, no. 1: 404. 10.1038/s42003-021-01854-3. PMC7994443 33767319

[psyp70414-bib-0046] Iber, C. , S. Ancoli‐Israel , A. Chesson , and S. Quan . 2007. “For the American Academy of Sleep Medicine.” In The AASM Manual for the Scoring of Sleep and Associated Events: Rules, Terminology and Technical Specifications. American Academy of Sleep Medicine.

[psyp70414-bib-0047] Johns, M. W. 1991. “A New Method for Measuring Daytime Sleepiness: The Epworth Sleepiness Scale.” Sleep 14, no. 6: 540–545. 10.1093/sleep/14.6.540. 1798888

[psyp70414-bib-0048] Jones, B. J. , and R. M. C. Spencer . 2019. “Sleep Preserves Subjective and Sympathetic Emotional Response of Memories.” Neurobiology of Learning and Memory 166: 107096. 10.1016/j.nlm.2019.107096. PMC7927201 31585163

[psyp70414-bib-0049] Kensinger, E. A. 2004. “Remembering Emotional Experiences: The Contribution of Valence and Arousal.” Reviews in the Neurosciences 15, no. 4: 241–252. 10.1515/REVNEURO.2004.15.4.241. 15526549

[psyp70414-bib-0050] Kleinknecht, R. A. , and R. M. Thorndike . 1990. “The Mutilation Questionnaire as a Predictor of Blood/Injury Fear and Fainting.” Behaviour Research and Therapy 28, no. 5: 429–437. 10.1016/0005-7967(90)90163-D. 2256902

[psyp70414-bib-0051] Klinzing, J. G. , N. Niethard , and J. Born . 2019. “Mechanisms of Systems Memory Consolidation During Sleep.” Nature Neuroscience 22, no. 10: 1598–1610. 10.1038/s41593-019-0467-3. 31451802

[psyp70414-bib-0052] LaBar, K. S. , and R. Cabeza . 2006. “Cognitive Neuroscience of Emotional Memory.” Nature Reviews Neuroscience 7, no. 1: 54–64. 10.1038/nrn1825. 16371950

[psyp70414-bib-0053] Lara‐Carrasco, J. , T. A. Nielsen , E. Solomonova , K. Levrier , and A. Popova . 2009. “Overnight Emotional Adaptation to Negative Stimuli Is Altered by REM Sleep Deprivation and Is Correlated With Intervening Dream Emotions.” Journal of Sleep Research 18, no. 2: 178–187. 10.1111/j.1365-2869.2008.00709.x. 19645964

[psyp70414-bib-0054] Lehmann, M. , T. Schreiner , E. Seifritz , and B. Rasch . 2016. “Emotional Arousal Modulates Oscillatory Correlates of Targeted Memory Reactivation During NREM, but Not REM Sleep.” Scientific Reports 6, no. 1: 39229. 10.1038/srep39229. PMC5159847 27982120

[psyp70414-bib-0055] Lipinska, G. , H. Austin , J. R. Moonsamy , et al. 2022. “Preferential Consolidation of Emotional Reactivity During Sleep: A Systematic Review and Meta‐Analysis.” Frontiers in Behavioral Neuroscience 16: 976047. 10.3389/fnbeh.2022.976047. PMC9578377 36268469

[psyp70414-bib-0056] Lipinska, G. , B. Stuart , K. G. F. Thomas , D. S. Baldwin , and E. Bolinger . 2019. “Preferential Consolidation of Emotional Memory During Sleep: A Meta‐Analysis.” Frontiers in Psychology 10: 1014. 10.3389/fpsyg.2019.01014. PMC6524658 31133940

[psyp70414-bib-0057] Lipinska, G. , and K. G. F. Thomas . 2019. “The Interaction of REM Fragmentation and Night‐Time Arousal Modulates Sleep‐Dependent Emotional Memory Consolidation.” Frontiers in Psychology 10: 1766. 10.3389/fpsyg.2019.01766. PMC6688536 31428021

[psyp70414-bib-0058] Lüdecke, D. , M. Ben‐Shachar , I. Patil , and D. Makowski . 2020. “Extracting, Computing and Exploring the Parameters of Statistical Models Using R.” Journal of Open Source Software 5, no. 53: 2445. 10.21105/joss.02445.

[psyp70414-bib-0059] Marchewka, A. , Ł. Żurawski , K. Jednoróg , and A. Grabowska . 2014. “The Nencki Affective Picture System (NAPS): Introduction to a Novel, Standardized, Wide‐Range, High‐Quality, Realistic Picture Database.” Behavior Research Methods 46, no. 2: 596–610. 10.3758/s13428-013-0379-1. PMC4030128 23996831

[psyp70414-bib-0060] Marshall, L. , and J. Born . 2007. “The Contribution of Sleep to Hippocampus‐Dependent Memory Consolidation.” Trends in Cognitive Sciences 11, no. 10: 442–450. 10.1016/j.tics.2007.09.001. 17905642

[psyp70414-bib-0061] McCullagh, P. 1980. “Regression Models for Ordinal Data.” Journal of the Royal Statistical Society, Series B: Statistical Methodology 42, no. 2: 109–127. 10.1111/j.2517-6161.1980.tb01109.x.

[psyp70414-bib-0062] McDevitt, E. A. , K. M. Rowe , M. Brady , K. A. Duggan , and S. C. Mednick . 2014. “The Benefit of Offline Sleep and Wake for Novel Object Recognition.” Experimental Brain Research 232, no. 5: 1487–1496. 10.1007/s00221-014-3830-3. 24504196

[psyp70414-bib-0063] McGaugh, J. L. 2004. “The Amygdala Modulates the Consolidation of Memories of Emotionally Arousing Experiences.” Annual Review of Neuroscience 27, no. 1: 1–28. 10.1146/annurev.neuro.27.070203.144157. 15217324

[psyp70414-bib-0064] Mickley Steinmetz, K. R. , D. R. Addis , and E. A. Kensinger . 2010. “The Effect of Arousal on the Emotional Memory Network Depends on Valence.” NeuroImage 53, no. 1: 318–324. 10.1016/j.neuroimage.2010.06.015. PMC3063443 20542121

[psyp70414-bib-0065] Natale, V. , M. J. Esposito , M. Martoni , and M. Fabbri . 2006. “Validity of the Reduced Version of the Morningness‐Eveningness Questionnaire.” Sleep and Biological Rhythms 4, no. 1: 72–74. 10.1111/j.1479-8425.2006.00192.x.

[psyp70414-bib-0066] Nishida, M. , J. Pearsall , R. L. Buckner , and M. P. Walker . 2009. “REM Sleep, Prefrontal Theta, and the Consolidation of Human Emotional Memory.” Cerebral Cortex 19, no. 5: 1158–1166. 10.1093/cercor/bhn155. PMC2665156 18832332

[psyp70414-bib-0067] Pace‐Schott, E. F. , E. Shepherd , R. M. C. Spencer , et al. 2011. “Napping Promotes Inter‐Session Habituation to Emotional Stimuli.” Neurobiology of Learning and Memory 95, no. 1: 24–36. 10.1016/j.nlm.2010.10.006. PMC3032361 20969968

[psyp70414-bib-0068] Payne, J. D. , and E. A. Kensinger . 2010. “Sleep's Role in the Consolidation of Emotional Episodic Memories.” Current Directions in Psychological Science 19, no. 5: 290–295. 10.1177/0963721410383978.

[psyp70414-bib-0069] Payne, J. D. , E. A. Kensinger , E. J. Wamsley , et al. 2015. “Napping and the Selective Consolidation of Negative Aspects of Scenes.” Emotion 15, no. 2: 176–186. 10.1037/a0038683. PMC5846328 25706830

[psyp70414-bib-0070] Peirce, J. , J. R. Gray , S. Simpson , et al. 2019. “PsychoPy2: Experiments in Behavior Made Easy.” Behavior Research Methods 51, no. 1: 195–203. 10.3758/s13428-018-01193-y. PMC6420413 30734206

[psyp70414-bib-0071] Perakakis, P. , M. Joffily , M. Taylor , P. Guerra , and J. Vila . 2010. “KARDIA: A Matlab Software for the Analysis of Cardiac Interbeat Intervals.” Computer Methods and Programs in Biomedicine 98, no. 1: 83–89. 10.1016/j.cmpb.2009.10.002. 19896748

[psyp70414-bib-0072] Prehn‐Kristensen, A. , I. Molzow , A. Förster , et al. 2017. “Memory Consolidation of Socially Relevant Stimuli During Sleep in Healthy Children and Children With Attention‐Deficit/Hyperactivity Disorder and Oppositional Defiant Disorder: What You Can See in Their Eyes.” Biological Psychology 123: 196–204. 10.1016/j.biopsycho.2016.12.017. 28049026

[psyp70414-bib-0073] R Core Team . 2023. R: A Language and Environment for Statistical Computing. R Foundation for Statistical Computing. https://www.R‐project.org/.

[psyp70414-bib-0074] Rasch, B. , and J. Born . 2013. “About Sleep's Role in Memory.” Physiological Reviews 93, no. 2: 681–766. 10.1152/physrev.00032.2012. PMC3768102 23589831

[psyp70414-bib-0092] Reinhold, F. L. , E. J. Van Someren , and M. Kindt . 2025. “Nighttime is the Right Time: The Time Interval to Overnight Sleep Following an Embarrassing Experience Does Not Influence Long‐Term Emotional Responses to its Reactivated Episode.” SLEEP Advances 6, no. 2: zpaf029. 10.1093/sleepadvances/zpaf029. PMC12168128 40524940

[psyp70414-bib-0075] Schad, D. J. , S. Vasishth , S. Hohenstein , and R. Kliegl . 2020. “How to Capitalize on a Priori Contrasts in Linear (Mixed) Models: A Tutorial.” Journal of Memory and Language 110: 104038. 10.1016/j.jml.2019.104038.

[psyp70414-bib-0076] Schäfer, S. K. , B. E. Wirth , M. Staginnus , N. Becker , T. Michael , and M. R. Sopp . 2020. “Sleep's Impact on Emotional Recognition Memory: A Meta‐Analysis of Whole‐Night, Nap, and REM Sleep Effects.” Sleep Medicine Reviews 51: 101280. 10.1016/j.smrv.2020.101280. 32179376

[psyp70414-bib-0077] Stark, S. M. , C. B. Kirwan , and C. E. L. Stark . 2019. “Mnemonic Similarity Task: A Tool for Assessing Hippocampal Integrity.” Trends in Cognitive Sciences 23, no. 11: 938–951. 10.1016/j.tics.2019.08.003. PMC6991464 31597601

[psyp70414-bib-0078] Tempesta, D. , V. Socci , L. De Gennaro , and M. Ferrara . 2018. “Sleep and Emotional Processing.” Sleep Medicine Reviews 40: 183–195. 10.1016/j.smrv.2017.12.005. 29395984

[psyp70414-bib-0079] van der Helm, E. , J. Yao , S. Dutt , V. Rao , J. M. Saletin , and M. P. Walker . 2011. “REM Sleep Depotentiates Amygdala Activity to Previous Emotional Experiences.” Current Biology 21, no. 23: 2029–2032. 10.1016/j.cub.2011.10.052. PMC3237718 22119526

[psyp70414-bib-0080] Vandewalle, G. , B. Middleton , S. M. W. Rajaratnam , et al. 2007. “Robust Circadian Rhythm in Heart Rate and Its Variability: Influence of Exogenous Melatonin and Photoperiod.” Journal of Sleep Research 16, no. 2: 148–155. 10.1111/j.1365-2869.2007.00581.x. 17542944

[psyp70414-bib-0081] Vignatelli, L. , G. Plazzi , A. Barbato , et al. 2003. “Italian Version of the Epworth Sleepiness Scale: External Validity.” Neurological Sciences 23, no. 6: 295–300. 10.1007/s100720300004. 12624716

[psyp70414-bib-0082] Viselli, L. , F. Salfi , F. Naccarato , et al. 2025. “Experimentally Induced REM Sleep Fragmentation Affects Psychophysiological Habituation to Emotional Stimuli.” Sleep 49, no. 4: zsaf409. 10.1093/sleep/zsaf409. PMC13089512 41432255

[psyp70414-bib-0083] Wagner, U. 2002. “Changes in Emotional Responses to Aversive Pictures Across Periods Rich in Slow‐Wave Sleep Versus Rapid Eye Movement Sleep.” Psychosomatic Medicine 64, no. 4: 627–634. 10.1097/01.psy.0000021940.35402.51. 12140353

[psyp70414-bib-0084] Wagner, U. , S. Gais , and J. Born . 2001. “Emotional Memory Formation Is Enhanced Across Sleep Intervals With High Amounts of Rapid Eye Movement Sleep.” Learning & Memory 8, no. 2: 112–119. 10.1101/lm.36801. PMC311359 11274257

[psyp70414-bib-0085] Wagner, U. , M. Hallschmid , B. Rasch , and J. Born . 2006. “Brief Sleep After Learning Keeps Emotional Memories Alive for Years.” Biological Psychiatry 60, no. 7: 788–790. 10.1016/j.biopsych.2006.03.061. 16806090

[psyp70414-bib-0086] Walker, M. P. , and E. Van Der Helm . 2009. “Overnight Therapy? The Role of Sleep in Emotional Brain Processing.” Psychological Bulletin 135, no. 5: 731–748. 10.1037/a0016570. PMC2890316 19702380

[psyp70414-bib-0087] Wassing, R. , J. S. Benjamins , L. M. Talamini , F. Schalkwijk , and E. J. W. Van Someren . 2019. “Overnight Worsening of Emotional Distress Indicates Maladaptive Sleep in Insomnia.” Sleep 42, no. 4: zsy268. 10.1093/sleep/zsy268. 30590834

[psyp70414-bib-0088] Werner, G. G. , M. Schabus , J. Blechert , and F. H. Wilhelm . 2021. “Differential Effects of REM Sleep on Emotional Processing: Initial Evidence for Increased Short‐Term Emotional Responses and Reduced Long‐Term Intrusive Memories.” Behavioral Sleep Medicine 19, no. 1: 83–98. 10.1080/15402002.2020.1713134. 31971007

[psyp70414-bib-0089] Wilhelm, I. , Y. Azza , K. Brennwald , Y. Ehrt‐Schäfer , E. Seifritz , and B. Kleim . 2021. “Investigating the Effect of a Nap Following Experimental Trauma on Analogue PTSD Symptoms.” Scientific Reports 11, no. 1: 4710. 10.1038/s41598-021-83838-1. PMC7907077 33633161

[psyp70414-bib-0090] Yuksel, C. , D. Denis , J. Coleman , et al. 2025. “Both Slow Wave and Rapid Eye Movement Sleep Contribute to Emotional Memory Consolidation.” Communications Biology 8, no. 1: 485. 10.1038/s42003-025-07868-5. PMC11930935 40123003

[psyp70414-bib-0091] Zeng, S. , X. Lin , J. Wang , and X. Hu . 2021. “Sleep's Short‐Term Memory Preservation and Long‐Term Affect Depotentiation Effect in Emotional Memory Consolidation: Behavioral and EEG Evidence.” Sleep 44, no. 11: zsab155. 10.1093/sleep/zsab155. 34153105

