# Supplementary material for: Emotional Processing in a Nap Paradigm: The Role of Sleep Stages in Memory and Reactivity
Source: Psychophysiology. 2026 Oct 15;63(10):e70414. doi: 10.1111/psyp.70414 (PMC13673111; doi:10.1111/psyp.70414)
Supplement: Supplementary file 1 [file PSYP-63-e70414-s001.docx]

**Emotional processing in a nap paradigm: the role of sleep stages in memory and reactivity**

Costanza Colombo^1^*, Angie Baldassarri^1^*, Umberto Granziol^1^, Jessica Ongaro^1^, Lorenzo Viselli^2^, Michele Ferrara^2^, Aurora D’Atri^2^, Nicola Cellini^1^**

^1^Department of General Psychology, University of Padua, Padua, Italy

^2^Department of Biotechnological and Applied Clinical Sciences, University of L’Aquila, L’Aquila, Italy

* These authors equally contributed to the manuscript

** Corresponding Author

Corresponding author:

Nicola Cellini, Department of General Psychology, University of Padova

Via Venezia 8, 35131, Padova (IT). Phone: +390498276603 [nicola.cellini@unipd.it](mailto:nicola.cellini@unipd.it)

ORCID:

Costanza Colombo: 0009-0005-8049-0381

Angie Baldassarri: 0009-0000-7489-1203

Umberto Granziol: 0000-0002-6286-6569

Jessica Ongaro: 0009-0002-8294-3936

Lorenzo Viselli: 0000-0003-3939-1101

Michele Ferrara: 0000-0003-2304-7576

Aurora D’Atri: 0000-0003-3650-0683

Nicola Cellini: 0000-0003-0306-4408

1. **Memory performance**

The linear mixed model conducted to assess memory performance at T0, with Group and Type of image as fixed effects and subjects as random effect, showed a difference in d-prime between negative and neutral images, with higher performance for negative images compared to neutral ones (β = -0.22, SE = 0.06, t = -3.75, p < .001, β* = -0.46). Full model results can be found in Table S1, followed by full model results of Negative (Table S2) and Neutral (Table S3) images.

**Table S1.** Full model results of d-prime analysis at T0

| **d-prime T0** | | | | | | |
| --- | --- | --- | --- | --- | --- | --- |
| *Coefficient* | *Estimate* | | *SE* | *CI (95%)* | *t* | *p* |
| (Intercept) | 2.81 | | 0.05 | 2.70 – 2.91 | 51.69 | **.001** |
| Group1 | -0.09 | | 0.07 | -0.24 – 0.05 | -1.30 | .195 |
| Group2 | -0.05 | | 0.03 | -0.12 – 0.02 | -1.37 | .172 |
| TypeNeutral | -0.22 | | 0.06 | -0.34 – -0.10 | -3.75 | **.001** |
| Group1 × TypeNeutral | 0.09 | | 0.08 | -0.07 – 0.24 | 1.09 | .279 |
| Group2 × TypeNeutral | -0.01 | | 0.04 | -0.09 – 0.06 | -0.39 | .696 |
| **Random Effects** | | | | | | |
| σ^2^ | | 0.12 | | | | |
| τ_00_ _ID_ | | 0.09 | | | | |
| ICC | | 0.41 | | | | |
| N _ID_ | | 80 | | | | |
| Observations | | 160 | | | | |
| Marginal R^2^ / Conditional R^2^ | | 0.095 / 0.468 | | | | |

Notes. **TypeNeutral =** Neutral – Negative; **Group1 =** REM – No-REM; **Group2** = Wake $\frac{No-REM+REM}{2}$; **SE** = Standard Error; **CI (95%)** = 95% Confidence Interval. Random effects: σ2 = residual variance (variance not explained by the model); τ00 ID = random intercept variance for participants (baseline difference between participants); ICC = intraclass correlation coefficient, representing the proportion of variance due to inter-participant differences; N ID = number of participants. Marginal R2 / Conditional R2 = model variance explained by fixed effects / model variance explained including random intercept

**Table S2.** Full model results of d-prime analysis on negative images

| **d-prime Negative images** | | | | | | |
| --- | --- | --- | --- | --- | --- | --- |
| *Coefficient* | *Estimate* | *SE* | *CI (95%)* | *t* | *p* |  |
| (Intercept) | 2.43 | 0.05 | 2.33 – 2.53 | 46.55 | **.001** |  |
| Group1 | 0.01 | 0.07 | -0.13 – 0.15 | 0.15 | .882 |  |
| Group2 | -0.08 | 0.03 | -0.14 – -0.01 | -2.32 | **.023** |  |
| Session2-1 | -0.38 | 0.07 | -0.53 – -0.24 | -5.37 | **.001** |  |
| Session3-2 | -0.36 | 0.07 | -0.50 – -0.22 | -4.97 | **.001** |  |
| Group1 × Session2-1 | 0.16 | 0.10 | -0.03 – 0.35 | 1.64 | .103 |  |
| Group2 × Session2-1 | -0.03 | 0.05 | -0.12 – 0.06 | -0.66 | .511 |  |
| Group1 × Session3-2 | 0.00 | 0.10 | -0.19 – 0.19 | 0.02 | .985 |  |
| Group2 × Session3-2 | -0.03 | 0.05 | -0.12 – 0.06 | -0.62 | .536 |  |
| **Random Effects** | | | | | |  |
| σ^2^ | 0.18 | | | | |  |
| τ_00_ _ID_ | 0.13 | | | | |  |
| ICC | 0.42 | | | | |  |
| N _ID_ | 80 | | | | |  |
| Observations | 237 | | | | |  |
| Marginal R^2^ / Conditional R^2^ | 0.270 / 0.577 | | | | |  |

Notes. **Group1 =** REM – No-REM; **Group2** = Wake – $\frac{No-REM+REM}{2}$; **Session2-1** = T1 – T0; **Session3-2** = T2 – T1; **SE** = Standard Error; **CI (95%)** = 95% Confidence Interval

**Table S3.** Full model results of d-prime analysis on neutral images

| **d-prime Neutral images** | | | | | |
| --- | --- | --- | --- | --- | --- |
| *Coefficient* | *Estimate* | *SE* | *CI (95%)* | *t* | *p* |
| (Intercept) | 2.21 | 0.06 | 2.10 – 2.33 | 39.08 | **.001** |
| Group1 | 0.01 | 0.08 | -0.14 – 0.16 | 0.09 | .927 |
| Group2 | -0.08 | 0.04 | -0.15 – -0.01 | -2.24 | **.028** |
| Session2-1 | -0.33 | 0.06 | -0.44 – -0.21 | -5.63 | **.001** |
| Session3-2 | -0.46 | 0.06 | -0.58 – -0.35 | -7.86 | **.001** |
| Group1 × Session2-1 | 0.02 | 0.08 | -0.14 – 0.17 | 0.20 | .839 |
| Group2 × Session2-1 | -0.08 | 0.04 | -0.15 – -0.01 | -2.21 | **.029** |
| Group1× Session3-2 | 0.02 | 0.08 | -0.14 – 0.17 | 0.20 | .844 |
| Group2 × Session3-2 | 0.11 | 0.04 | 0.03 – 0.18 | 2.88 | **.005** |
| **Random Effects** | | | | | |
| σ^2^ | 0.12 | | | | |
| τ_00_ _ID_ | 0.19 | | | | |
| ICC | 0.61 | | | | |
| N _ID_ | 80 | | | | |
| Observations | 237 | | | | |
| Marginal R^2^ / Conditional R^2^ | 0.279 / 0.718 | | | | |

Notes. **Group1 =** REM – No-REM; **Group2** = Wake – $\frac{No-REM+REM}{2}$; **Session2-1** = T1 – T0; **Session3-2** = T2 – T1; **SE** = Standard Error; **CI (95%)** = 95% Confidence Interval

2. **Subjective reactivity**
   2.1. **Arousal**

The cumulative link model conducted on Arousal to assess changes in subjective arousal at T0, with Group and Type as fixed effects, and subjects as random effect, revealed a difference between negative and neutral images, indicating that neutral images were rated with lower scores compared to negative ones (i.e., neutral images were evaluated as less arousing compared to negative images) (log-odds = -4.75, SE = 0.13, z = -36.22, p <.001, OR = 0.01). Moreover, the difference between negative and neutral images varied across groups, with a stronger difference between neutral and negative images in the REM group compared to the No-REM group (log-odds = -0.32, SE = 0.12, z = -2.73, p = .006, OR = 0.73), as represented in Figure S1a. Furthermore, the difference between negative and neutral images was higher in the Wake group than in the Nap group (log-odds = -0.22, SE = 0.06, z = -3.94, p < .001, OR = 0.80) as represented in Figure S1b. Full model results can be found in Table S4, followed by the results of Negative (Table S5) and Neutral (Table S6).

**Table S4.** Full model results on Arousal at T0

| **Arousal T0** | | | | | |
| --- | --- | --- | --- | --- | --- |
| *Predictors* | *Log-Odds* | *SE* | *CI* | *z* | *p* |
| TypeNeutral | -4.75 | 0.13 | -5.01 – -4.50 | -36.22 | **<.001** |
| Group1 | 0.30 | 0.25 | -0.19 – 0.80 | 1.19 | .234 |
| Group2 | 0.06 | 0.12 | -0.17 – 0.29 | 0.51 | .609 |
| TypeNeutral × Group1 | -0.32 | 0.12 | -0.55 – -0.09 | -2.73 | **.006** |
| TypeNeutral × Group2 | -0.22 | 0.06 | -0.33 – -0.11 | -3.94 | **<.001** |
| **Random Effects** | | | | | |
| σ^2^ | 3.29 | | | | |
| τ_00_ _ID_ | 2.33 | | | | |
| ICC | 0.41 | | | | |
| N _ID_ | 80 | | | | |
| Observations | 2240 | | | | |
| Marginal R^2^ / Conditional R^2^ | 0.515 / 0.716 | | | | |

Notes. **TypeNeutral =** Neutral – Negative; **Group1 =** REM – No-REM; **Group2** = Wake – $\frac{No-Rem+Rem}{2}$; **SE** = Standard Error; **CI (95%)** = 95% Confidence Interval

**Figure S1.** **Predicted probability of Arousal at T0.** **(a)** Comparison between negative and neutral images for No-REM and REM groups. **(b)** Comparison between negative and neutral images for Nap and Wake groups.

Each dot represents the mean probability estimated by the model for each ordinal level. Error bars represent the standard error estimated by the model. Numbers from 1 to 9 above the plot indicate ordinal responses on the Self-Assessment Manikin, with 1 corresponding to low arousal levels and 9 to high arousal levels.

| **Arousal Negative images** | | | | | |
| --- | --- | --- | --- | --- | --- |
| *Predictors* | *Log-Odds* | *SE* | *CI* | *z* | *p* |
| Group1 | 0.08 | 0.43 | -0.78 – 0.93 | 0.18 | .860 |
| Group2 | 0.20 | 0.20 | -0.20 – 0.60 | 0.97 | .332 |
| Session2-1 | -0.53 | 0.08 | -0.69 – -0.37 | -6.44 | **<.001** |
| Session3-2 | -0.08 | 0.08 | -0.24 – 0.08 | -0.99 | .322 |
| Group1 × Session2-1 | -0.31 | 0.11 | -0.53 – -0.10 | -2.86 | **.004** |
| Group2× Session2-1 | 0.17 | 0.05 | 0.07 – 0.27 | 3.26 | **.001** |
| Group1 × Session3-2 | -0.15 | 0.11 | -0.36 – 0.07 | -1.34 | .180 |
| Group2 × Session3-2 | -0.06 | 0.05 | -0.16 – 0.04 | -1.18 | .239 |
| **Random Effects** | | | | | |
| σ^2^ | 3.29 | | | | |
| τ_00_ _ID_ | 7.47 | | | | |
| ICC | 0.69 | | | | |
| N _ID_ | 80 | | | | |
| Observations | 3318 | | | | |
| Marginal R^2^ / Conditional R^2^ | 0.016 / 0.699 | | | | |

**Table S5.** Full model results on Arousal on negative images

Notes. **Group1 =** REM – No-REM; **Group2** = Wake – $\frac{No-Rem+Rem}{2}$; **Session2-1** = T1 – T0; **Session3-2** = T2 – T1; **SE** = Standard Error; **CI (95%)** = 95% Confidence Interval

| **Arousal Neutral images** | | | | | |
| --- | --- | --- | --- | --- | --- |
| *Predictors* | *Log-Odds* | *SE* | *CI* | *z* | *p* |
| Group1 | 0.02 | 0.41 | -0.78 – 0.82 | 0.05 | .959 |
| Group2 | -0.19 | 0.19 | -0.57 – 0.19 | -0.99 | .322 |
| Session2-1 | -0.58 | 0.10 | -0.78 – -0.38 | -5.75 | **<.001** |
| Session3-2 | 0.01 | 0.10 | -0.19 – 0.20 | 0.06 | .956 |
| Group1 × Session2-1 | -0.06 | 0.14 | -0.32 – 0.21 | -0.43 | .670 |
| Group2 × Session2-1 | 0.17 | 0.06 | 0.05 – 0.29 | 2.68 | **.007** |
| Group1 × Session3-2 | 0.12 | 0.14 | -0.15 – 0.38 | 0.85 | .398 |
| Group2 × Session3-2 | -0.21 | 0.06 | -0.33 – -0.08 | -3.26 | **.001** |
| **Random Effects** | | | | | |
| σ^2^ | 3.29 | | | | |
| τ_00_ _ID_ | 6.37 | | | | |
| ICC | 0.66 | | | | |
| N _ID_ | 80 | | | | |
| Observations | 3318 | | | | |
| Marginal R^2^ / Conditional R^2^ | 0.017 / 0.665 | | | | |

**Table S6.** Full model results on Arousal on neutral images

Notes. **Group1 =** REM – No-REM; **Group2** = Wake – $\frac{No-REM+REM}{2}$; **Session2-1** = T1 – T0; **Session3-2** = T2 – T1; **SE** = Standard Error; **CI (95%)** = 95% Confidence Interval

2.2. **Valence**

The cumulative link model conducted on Valence to assess changes in subjective valence at T0, with Group and Type as fixed effects, and subjects as random effect, revealed a difference between neutral and negative images, with negative images being more likely to receive lower ratings compared to neutral images (i.e., negative images were rated as less pleasant than neutral images) (log-odds = 5.89, SE = 0.17, z = 35.51, p <.001, OR = 3.62). Moreover, a difference between the No-REM and REM groups in the comparison between negative and neutral images was found, indicating that the difference between negative and neutral images was higher in the REM group than in the No-REM group (log-odds = 0.50, SE = 0.12, z = 4.32, p <.001, OR = 1.65), as represented in Figure S2a. Additionally, a difference between the Nap and Wake groups in the comparison between negative and neutral images was found, indicating that the negative images were rated with lower scores compared to neutral ones was higher in the Nap group than in Wake group (log-odds = -0.16, SE = 0.05, z = -2.93, p = .003, OR = 0.85), as represented in Figure S2b. Full model results can be found in Table S7, followed by the results of the negative model (Table S8) and the results of the neutral model (Table S9).

**Figure S2.** **Predicted probability of Valence at T0 a.** Comparison between negative and neutral images for No-REM and REM groups. **b.** Comparison between negative and neutral images for the Nap and Wake groups.

Each dot represents the mean probability estimated by the model for each ordinal level. Error bars represent the standard error estimated by the model. Numbers from 1 to 9 above the plot indicate ordinal responses on the Self-Assessment Manikin, with 1 corresponding to low valence (very negative image) and 9 to high valence (very positive image).

| **Valence T0** | | | | | |
| --- | --- | --- | --- | --- | --- |
| *Predictors* | *Log-Odds* | *SE* | *CI* | *z* | *p* |
| TypeNeutral | 5.89 | 0.17 | 5.57 – 6.22 | 35.51 | **<.001** |
| Group1 | -0.34 | 0.21 | -0.75 – 0.06 | -1.65 | .099 |
| Group2 | 0.03 | 0.10 | -0.16 – 0.22 | 0.32 | .747 |
| TypeNeutral × Group1 | 0.50 | 0.12 | 0.28 – 0.73 | 4.32 | **<.001** |
| TypeNeutral × Group2 | -0.16 | 0.05 | -0.27 – -0.05 | -2.93 | **.003** |
| **Random Effects** | | | | | |
| σ^2^ | 3.29 | | | | |
| τ_00_ _ID_ | 1.44 | | | | |
| ICC | 0.30 | | | | |
| N _ID_ | 80 | | | | |
| Observations | 2240 | | | | |
| Marginal R^2^ / Conditional R^2^ | 0.643 / 0.751 | | | | |

**Table S7.** Full model results on Valence at T0

Notes. **TypeNeutral =** Neutral – Negative; **Group1 =** REM – No-REM; **Group2** = Wake – $\frac{No-REM+REM}{2}$; **SE** = Standard Error; **CI (95%)** = 95% Confidence Interval

**Table S8.** Full model results on Valence on negative images

| **Valence Negative images** | | | | | |
| --- | --- | --- | --- | --- | --- |
| *Predictors* | *Log-Odds* | *SE* | *CI* | *Z* | *p* |
| Group1 | -0.25 | 0.32 | -0.88 – 0.37 | -0.79 | .428 |
| Group2 | -0.02 | 0.15 | -0.32 – 0.27 | -0.16 | .876 |
| Session2-1 | -0.12 | 0.09 | -0.30 – 0.06 | -1.35 | .177 |
| Session3-2 | 0.25 | 0.09 | 0.07 – 0.43 | 2.66 | **.008** |
| Group1 × Session2-1 | 0.07 | 0.12 | -0.17 – 0.31 | 0.57 | .572 |
| Group2 × Session2-1 | -0.05 | 0.06 | -0.17 – 0.06 | -0.96 | .337 |
| Group1 × Session3-2 | 0.50 | 0.13 | 0.25 – 0.74 | 3.97 | **<.001** |
| Group2 × Session3-2 | -0.16 | 0.06 | -0.27 – -0.04 | -2.70 | **.007** |
| **Random Effects** | | | | | |
| σ^2^ | 3.29 | | | | |
| τ_00_ _ID_ | 3.91 | | | | |
| ICC | 0.54 | | | | |
| N _ID_ | 80 | | | | |
| Observations | 3318 | | | | |
| Marginal R^2^ / Conditional R^2^ | 0.012 / 0.549 | | | | |

Notes. **Group1 =** REM – No-REM; **Group2** = Wake – $\frac{No-REM+REM}{2}$; **Session2-1** = T1 – T0; **Session3-2** = T2 – T1; **SE** = Standard Error; **CI (95%)** = 95% Confidence Interval

| **Valence Neutral images** | | | | | |
| --- | --- | --- | --- | --- | --- |
| *Predictors* | *Log-Odds* | *SE* | *CI* | *z* | *p* |
| Group1 | -0.02 | 0.20 | -0.42 – 0.37 | -0.12 | .906 |
| Group2 | -0.04 | 0.11 | -0.26 – 0.17 | -0.39 | .695 |
| Session2-1 | -0.27 | 0.09 | -0.45 – -0.09 | -2.97 | **.003** |
| Session3-2 | -0.27 | 0.09 | -0.45 – -0.08 | -2.86 | **.004** |
| Group1 × Session2-1 | -0.21 | 0.12 | -0.45 – 0.03 | -1.71 | .088 |
| Group2 × Session2-1 | 0.11 | 0.06 | -0.00 – 0.22 | 1.88 | .060 |
| Group1 × Session3-2 | 0.02 | 0.12 | -0.22 – 0.27 | 0.20 | .845 |
| Group2 × Session3-2 | 0.09 | 0.06 | -0.03 – 0.20 | 1.50 | .134 |
| **Random Effects** | | | | | |
| σ^2^ | 3.29 | | | | |
| τ_00_ _ID_ | 2.64 | | | | |
| ICC | 0.45 | | | | |
| N _ID_ | 80 | | | | |
| Observations | 3318 | | | | |
| Marginal R^2^ / Conditional R^2^ | 0.009 / 0.451 | | | | |
| AIC | 7470.351 | | | | |

**Table S9.** Full model results on Valence on neutral images

Notes. **Group1 =** REM – No-REM; **Group2** = Wake – $\frac{No-REM+REM}{2}$; **Session2-1** = T1 – T0; **Session3-2** = T2 – T1; **SE** = Standard Error; **CI (95%)** = 95% Confidence Interval

3. **Skin conductance response (SCR)**

The generalised linear mixed model conducted on SCR at T0 to assess changes in skin conductance response, with Group and Type as fixed effects, and subjects as random effect, revealed a difference between neutral and negative images, with a greater SCR for negative images compared to neutral ones (β = -0.25, SE = 0.04, t = -6.45, p <.001, β* = -0.25).

The model revealed the presence of outliers; therefore, the analysis was conducted excluding the influential observations.

The generalised linear mixed model conducted on SCR at T0, without the influential observations, with Group and Type as fixed effects, and subjects as random effect, revealed a difference between neutral and negative images, with a greater skin conductance response for negative images compared to neutral ones (β = -0.23, SE = 0.03, t = -6.89, p <.001, β* = -0.25). Moreover, a difference between the Wake and Nap groups in the contrast between neutral and negative images was found, indicating that the difference in SCR between neutral and negative images was greater in the Wake group compared to the Nap group (β = -0.06, SE = 0.02, t = -2.73, p = .007, β* = 0.06). Results from the two models, with and without outliers, can be found in Table S10. Nevertheless, we examined the case and found no reason to exclude the influential observation; therefore, we chose the first model.

**Table S10.** Full model results on SCR at T0 with and without outliers

| **Model with outliers** | | | | | | **Model without outliers** | | | | |
| --- | --- | --- | --- | --- | --- | --- | --- | --- | --- | --- |
| *Coefficient* | *Estimate* | *SE* | *CI (95%)* | *t* | *p* | *Estimate* | *SE* | *CI (95%)* | *t* | *p* |
| (Intercept) | -3.59 | 0.16 | -3.90 – -3.29 | -23.03 | **<.001** | -3.62 | 0.16 | -3.94 – -3.30 | -22.57 | **<.001** |
| Group1 | -0.09 | 0.21 | -0.50 – 0.33 | -0.41 | .681 | -0.11 | 0.22 | -0.54 – 0.32 | -0.52 | .606 |
| Group2 | 0.02 | 0.10 | -0.17 – 0.21 | 0.20 | .841 | 0.01 | 0.10 | -0.18 – 0.21 | 0.14 | .888 |
| TypeNeutral | -0.25 | 0.04 | -0.32 – -0.17 | -6.45 | **<.001** | -0.23 | 0.03 | -0.29 – -0.16 | -6.89 | **<.001** |
| Group1 × TypeNeutral | -0.05 | 0.05 | -0.15 – 0.05 | -0.95 | .344 | -0.05 | 0.04 | -0.14 – 0.04 | -1.05 | .297 |
| Group2 × TypeNeutral | 0.04 | 0.02 | -0.01 – 0.09 | 1.76 | .081 | 0.06 | 0.02 | 0.02 – 0.10 | 2.73 | **.007** |
| **Random Effects** | | | | | | | | | | |
| σ^2^ | 0.12 | | | | | 0.10 | | | | |
| τ_00_ | 0.59 _ID_ | | | | | 0.59 _ID_ | | | | |
| ICC | 0.83 | | | | | 0.85 | | | | |
| N | 78 _ID_ | | | | | 76 _ID_ | | | | |
| Observations | 156 | | | | | 150 | | | | |
| Marginal R^2^ / Conditional R^2^ | 0.032 / 0.832 | | | | | 0.035 / 0.855 | | | | |
| AIC | -958.652 | | | | | -946.340 | | | | |

Notes. **TypeNeutral =** Neutral – Negative; **Group1 =** REM – No-REM; **Group2** = Wake – $\frac{No-REM+REM}{2}$; **SE** = Standard Error; **CI (95%)** = 95% Confidence Interval

**Table S11.** Full model results on SCR for negative images

| **SCR Negative images** | | | | | |
| --- | --- | --- | --- | --- | --- |
| *Predictors* | *Estimates* | *SE* | *CI* | *t* | *p* |
| (Intercept) | -3.76 | 0.13 | -4.01 – -3.50 | -29.22 | **<.001** |
| Group1 | -0.08 | 0.17 | -0.42 – 0.27 | -0.44 | .662 |
| Group2 | 0.01 | 0.08 | -0.14 – 0.17 | 0.18 | .857 |
| Session2-1 | -0.41 | 0.11 | -0.63 – -0.18 | -3.62 | **<.001** |
| Session3-2 | -0.10 | 0.11 | -0.33 – 0.12 | -0.91 | .366 |
| Group1 × Session2-1 | -0.10 | 0.15 | -0.40 – 0.20 | -0.68 | .500 |
| Group2 × Session2-1 | 0.05 | 0.07 | -0.09 – 0.19 | 0.69 | .489 |
| Group1 × Session3-2 | 0.05 | 0.16 | -0.26 – 0.35 | 0.29 | .772 |
| Group2 × Session3-2 | -0.04 | 0.07 | -0.18 – 0.10 | -0.57 | .572 |
| **Random Effects** | | | | | |
| σ^2^ | 0.33 | | | | |
| τ_00_ _ID_ | 0.48 | | | | |
| ICC | 0.59 | | | | |
| N _ID_ | 78 | | | | |
| Observations | 229 | | | | |
| Marginal R^2^ / Conditional R^2^ | 0.059 / 0.614 | | | | |
| AIC | -1148.968 | | | | |

Notes. **Group1 =** REM – No-REM; **Group2** = Wake – $\frac{No-REM+REM}{2}$; **Session2-1** = T1 – T0; **Session3-2** = T2 – T1; **SE** = Standard Error; **CI (95%)** = 95% Confidence Interval

**Table S12.** Full model results on SCR for neutral images

| **SCR Neutral** | | | | | |
| --- | --- | --- | --- | --- | --- |
| *Coefficient* | *Estimate* | *SE* | *CI (95%)* | *t* | *p* |
| (Intercept) | -3.94 | 0.13 | -4.20 – -3.68 | -29.53 | **<.001** |
| Group1 | -0.17 | 0.18 | -0.53 – 0.18 | -0.97 | .333 |
| Group2 | 0.05 | 0.08 | -0.11 – 0.22 | 0.63 | .529 |
| Session2-1 | -0.32 | 0.11 | -0.53 – -0.11 | -2.98 | **.003** |
| Session3-2 | -0.06 | 0.11 | -0.28 – 0.16 | -0.50 | .618 |
| Group1 × Session2-1 | -0.15 | 0.15 | -0.44 – 0.13 | -1.04 | .298 |
| Group2 × Session2-1 | 0.00 | 0.07 | -0.13 – 0.14 | 0.06 | .949 |
| Group1 × Session3-2 | 0.07 | 0.15 | -0.23 – 0.37 | 0.48 | .632 |
| Group2 × Session3-2 | 0.01 | 0.07 | -0.12 – 0.15 | 0.20 | .843 |
| **Random Effects** | | | | | |
| σ^2^ | 0.34 | | | | |
| τ_00_ _ID_ | 0.54 | | | | |
| ICC | 0.62 | | | | |
| N _ID_ | 78 | | | | |
| Observations | 229 | | | | |
| Marginal R^2^ / Conditional R^2^ | 0.053 / 0.635 | | | | |
| AIC | -1225.387 | | | | |

Notes. **Group1 =** No-REM – REM; **Group2** = Wake – $\frac{No-REM+REM}{2}$; **Session2-1** = T0 – T1; **Session3-2** = T1 – T2; **SE** = Standard Error; **CI (95%)** = 95% Confidence Interval

4. **Phasic cardiac response (∆PCR)**

The linear mixed model conducted on ∆PCR at T0 to assess cardiac response changes, with Group and Type of image as fixed effects and subjects as random effect, indicated a difference between neutral and negative images, with higher cardiac deceleration for negative images compared to neutral ones (β = 1.40, SE = 0.12, t = 11.77, p <.001, β* = 0.61). Moreover, a difference between the Wake and Nap groups across negative and neutral images was found. This result indicates an opposite trend for neutral and negative images, with Wake showing higher ∆PCR (i.e., a slight cardiac acceleration) than Nap for neutral images, but lower ∆PCR than Nap for negative images (i.e., greater cardiac deceleration) (β = 0.31, SE = 0.07, t = 4.11, p <.001, β* = 0.13). Full model results can be found in Table S13, followed by full model results of the negative (Table S14) and the neutral (Table S15) images.

**Table S13.** Full model results on ΔPCR analysis at T0

| **ΔPCR T0** | | | | | |
| --- | --- | --- | --- | --- | --- |
| *Coefficient* | *Estimate* | *SE* | *CI (95%)* | *t* | *p* |
| (Intercept) | -1.24 | 0.17 | -1.59 – -0.90 | -7.20 | **<.001** |
| Group1 | 0.22 | 0.23 | -0.24 – 0.67 | 0.93 | .355 |
| Group2 | -0.14 | 0.11 | -0.36 – 0.07 | -1.30 | .195 |
| TypeNeutral | 1.40 | 0.12 | 1.17 – 1.63 | 11.77 | **<.001** |
| Group1 × TypeNeutral | 0.08 | 0.16 | -0.23 – 0.39 | 0.51 | .612 |
| Group2 × TypeNeutral | 0.31 | 0.08 | 0.16 – 0.46 | 4.11 | **<.001** |
| **Random Effects** | | | | | |
| σ^2^ | 3.05 | | | | |
| τ_00_ _ID_ | 1.64 | | | | |
| ICC | 0.35 | | | | |
| N _ID_ | 80 | | | | |
| Observations | 960 | | | | |
| Marginal R^2^ / Conditional R^2^ | 0.129 / 0.433 | | | | |

Notes. **TypeNeutral =** Neutral – Negative; **Group1 =** REM – No-REM; **Group2** = Wake – $\frac{No-REM+REM}{2}$; **SE** = Standard Error; **CI (95%)** = 95% Confidence Interval

**Table S14.** Full model results on ΔPCR on negative images

| **∆PCR Negative images** | | | | | |
| --- | --- | --- | --- | --- | --- |
| *Coefficient* | *Estimate* | *SE* | *CI (95%)* | *t* | *p* |
| (Intercept) | -0.77 | 0.17 | -1.11 – -0.43 | -4.46 | **<.001** |
| Group1 | -0.26 | 0.20 | -0.65 – 0.14 | -1.30 | .198 |
| Group2 | -0.06 | 0.09 | -0.25 – 0.13 | -0.62 | .534 |
| Session2-1 | 0.46 | 0.25 | -0.07 – 0.98 | 1.83 | .086 |
| Session3-2 | 0.51 | 0.25 | -0.02 – 1.04 | 2.04 | .058 |
| Group1 × Session2-1 | -0.57 | 0.17 | -0.90 – -0.23 | -3.33 | **<.001** |
| Group2 × Session2-1 | 0.14 | 0.08 | -0.01 – 0.30 | 1.79 | .074 |
| Group1 × Session3-2 | -0.29 | 0.17 | -0.63 – 0.05 | -1.68 | .093 |
| Group2 × Session3-2 | -0.03 | 0.08 | -0.19 – 0.12 | -0.42 | .672 |
| **Random Effects** | | | | | |
| σ^2^ | 3.46 | | | | |
| τ_00_ _ID_ | 1.39 | | | | |
| τ_00_ _Session:Time_ | 0.14 | | | | |
| ICC | 0.31 | | | | |
| N _ID_ | 80 | | | | |
| N _Session_ | 3 | | | | |
| N _Time_ | 6 | | | | |
| Observations | 1422 | | | | |
| Marginal R^2^ / Conditional R^2^ | 0.054 / 0.344 | | | | |

Notes. **Group1 =** REM – No-REM; **Group2** = Wake – $\frac{No-REM+REM}{2}$; **Session2-1** = T1 – T0; **Session3-2** = T2 – T1; **SE** = Standard Error; **CI (95%)** = 95% Confidence Interval

**Table S15.** Full model results on ΔPCR on neutral images

| **∆PCR Neutral images** | | | | | |
| --- | --- | --- | --- | --- | --- |
| *Coefficient* | *Estimate* | *SE* | *CI (95%)* | *t* | *p* |
| (Intercept) | 0.46 | 0.14 | 0.18 – 0.74 | 3.29 | **.002** |
| Group1 | 0.27 | 0.16 | -0.04 – 0.58 | 1.73 | .087 |
| Group2 | 0.15 | 0.07 | 0.00 – 0.30 | 2.04 | **.045** |
| Session2-1 | 0.53 | 0.22 | 0.06 – 1.01 | 2.39 | **.030** |
| Session3-2 | -0.15 | 0.22 | -0.63 – 0.32 | -0.68 | .508 |
| Group1:Session2-1 | -0.04 | 0.16 | -0.35 – 0.26 | -0.27 | .791 |
| Group2:Session2-1 | 0.01 | 0.07 | -0.14 – 0.15 | 0.09 | .926 |
| Group1:Session3-2 | 0.00 | 0.16 | -0.31 – 0.32 | 0.03 | .975 |
| Group2:Session3-2 | -0.06 | 0.07 | -0.21 – 0.08 | -0.85 | .394 |
| **Random Effects** | | | | | |
| σ^2^ | 2.90 | | | | |
| τ_00_ _ID_ | 0.81 | | | | |
| τ_00_ _Session:Time_ | 0.11 | | | | |
| ICC | 0.24 | | | | |
| N _ID_ | 80 | | | | |
| N _Session_ | 3 | | | | |
| N _Time_ | 6 | | | | |
| Observations | 1422 | | | | |
| Marginal R^2^ / Conditional R^2^ | 0.035 / 0.267 | | | | |

Notes. **Group1 =** REM – No-REM; **Group2** = Wake – $\frac{No-REM+REM}{2}$; **Session2-1** = T1 – T0; **Session3-2** = T2 – T1; **SE** = Standard Error; **CI (95%)** = 95% Confidence Interval

5. **Controlling for the effect of total sleep time and time spent in N3 on emotional processing**

In light of the heterogeneity in total sleep time (TST) and time spent in N3 sleep stage (N3%) among participants, we conducted linear or generalised mixed models to assess whether TST and N3% could affect the variables considered in the study. We included only the Nap group and focused on sessions T0 and T1 only for negative images, both for reactivity measures (physiological and subjective) and for emotional memory.

**5.1. Memory performance**

The linear mixed model conducted on d-prime to assess changes in memory performance for negative images, with Session (T0, T1) and TST or N3% as fixed effects, and subjects as random effect, revealed a significant change across sessions T1 and T0, with a greater performance a T0 compared to T1 both for TST (β = -0.75, SE = 0.24, t = -3.12, p = .003) and N3% models (β = -0.51, SE = 0.14, t = -3.55, p = .001). No significant effect was revealed considering TST or N3% only and the interaction between TST or N3% and Sessions.

**5.2. PCR**

The linear mixed model conducted to assess changes in PCR for negative images, with Session (T0, T1) and TST as fixed effects, subjects as random effect and time of image presentation nested within Session, revealed no significant effects of Session, TST, or their interaction. For the model considering N3%, we observed a significant interaction between the Session and N3% (β = 0.03, SE = 0.01, t = 3.48, p < .001). Specifically, higher N3% was associated with lower PCR values at T0, whereas the association was weaker at T1, resulting in an increase of PCR values from T0 to T1 at higher N3% values. This finding is not consistent with the results observed in the REM vs No-REM group comparison.

**5.3. SCR**

The linear mixed model conducted to assess changes in SCR for negative images, with Session (T0, T1) and TST or N3% as fixed effects, and subjects as a random effect, revealed no significant effects of Session, TST or N3%, or their interaction.

**5.4. Subjective Valence and Arousal**

The linear model conducted on mean subjective ratings of Valence for negative images, with Session (T0, T1) and TST or N3% as fixed effects and subjects as random effects, revealed no significant change.

Otherwise, the linear model conducted on mean subjective rating of Arousal on negative images, with TST as fixed effect, reveals a significant interaction between TST and Session (β = –0.013, SE = 0.005, t = –2.18, p = .036), indicating that, as TST increases, the difference in the mean Arousal between T1 and T0 attenuates. No effects were found for Arousal ratings with N3% as fixed effect.

6. **Controlling for the REM percentage on emotional processing**

Analogously to the analyses conducted on TST, linear mixed-effects or generalised mixed models were performed to assess whether the percentage of REM sleep (REM%) affected the variables of interest. These analyses included only participants who reached REM sleep and focused on sessions T0 and T1, considering negative images exclusively, for both reactivity measures (physiological and subjective) and emotional memory.

**6.1. Memory performance**

The linear mixed model conducted on d-prime to assess changes in memory performance for negative images, with Session (T0, T1) and REM% as fixed effects, and subjects as random effect, revealed a significant change across sessions T1 and T0, with a greater performance a T0 compared to T1 (β = -0.56, SE = 0.23, t = -2.40, p = .027). No significant effect was revealed considering REM% only and the interaction between REM% and Sessions.

**6.2. SCR**

The linear mixed model conducted to assess changes on SCR for negative images, with Session (T0, T1) and REM% as fixed effects, and subjects as random effect, revealed no significant change across sessions or considering REM% only and the interaction between REM% and Sessions.

**6.3. PCR**

The linear mixed model conducted to assess changes on PCR for negative images, with Session (T0, T1) and REM% as fixed effects, subjects as random effect and Time of image presentation nested within Session, revealed no significant change across sessions or considering REM% only and the interaction between REM% and Sessions.

**6.4. Subjective Arousal and Valence**

Linear mixed model analyses of mean subjective ratings for negative images, with Session (T0, T1) and REM% as fixed effects and subjects as random factors, revealed no significant effects on either Valence or Arousal.

7. **Mean ratings for Arousal and Valence in the validation of the reactivity task**

Arousal and Valence ratings for each image used in the emotional reactivity task (Table S16), and mean ratings for each image set (Table S17) derived from a previous validation study with 25 university students (mean age ± SD, 24.68 ± 2.40, 14 males). In the study, participants underwent an online picture evaluation task through Pavlovia software (Open Science Tools, Nottingham, UK).

**Table S16.** Arousal and Valence ratings for each image

| **SET** | **Type of image** | **Images ID** | **Valence (mean)** | **Arousal (mean)** |
| --- | --- | --- | --- | --- |
| E1 | Neg | images/E1_Neg_1.JPG | 1.48 | 7.48 |
| E1 | Neg | images/E1_Neg_2.JPG | 1.8 | 6.84 |
| E1 | Neg | images/E1_Neg_3.JPG | 1.64 | 6.8 |
| E1 | Neg | images/E1_Neg_4.JPG | 2.04 | 6.56 |
| E1 | Neg | images/E1_Neg_5.JPG | 2.04 | 6.6 |
| E1 | Neg | images/E1_Neg_6.JPG | 2.16 | 6.64 |
| E1 | Neg | images/E1_Neg_7.JPG | 2.08 | 6.52 |
| E1 | Neg | images/E1_Neg_8.JPG | 2.2 | 6.16 |
| E1 | Neg | images/E1_Neg_9.JPG | 2.12 | 6.12 |
| E1 | Neg | images/E1_Neg_10.JPG | 2.36 | 6.04 |
| E1 | Neg | images/E1_Neg_11.JPG | 3.12 | 6 |
| E1 | Neg | images/E1_Neg_12.JPG | 2.44 | 5.84 |
| E1 | Neg | images/E1_Neg_13.JPG | 2.28 | 5.96 |
| E1 | Neg | images/E1_Neg_14.JPG | 2.32 | 5.56 |
| E1 | Neu | images/E1_Neu_1.JPG | 5.88 | 3.04 |
| E1 | Neu | images/E1_Neu_2.JPG | 5.4 | 2.76 |
| E1 | Neu | images/E1_Neu_3.JPG | 5.96 | 2.72 |
| E1 | Neu | images/E1_Neu_4.JPG | 6.04 | 2.68 |
| E1 | Neu | images/E1_Neu_5.JPG | 5.64 | 2.64 |
| E1 | Neu | images/E1_Neu_6.JPG | 6.12 | 2.64 |
| E1 | Neu | images/E1_Neu_7.JPG | 5.44 | 2.6 |
| E1 | Neu | images/E1_Neu_8.JPG | 5.76 | 2.6 |
| E1 | Neu | images/E1_Neu_9.JPG | 5.96 | 2.56 |
| E1 | Neu | images/E1_Neu_10.JPG | 5.88 | 2.4 |
| E1 | Neu | images/E1_Neu_11.JPG | 5.48 | 2.24 |
| E1 | Neu | images/E1_Neu_12.JPG | 5.4 | 2.04 |
| E1 | Neu | images/E1_Neu_13.JPG | 5.6 | 2.04 |
| E1 | Neu | images/E1_Neu_14.JPG | 5.48 | 1.8 |
| E2 | Neg | images/E2_Neg_1.JPG | 1.36 | 7.36 |
| E2 | Neg | images/E2_Neg_2.JPG | 1.36 | 7.08 |
| E2 | Neg | images/E2_Neg_3.JPG | 1.64 | 6.76 |
| E2 | Neg | images/E2_Neg_4.JPG | 1.8 | 6.68 |
| E2 | Neg | images/E2_Neg_5.JPG | 2.16 | 6.6 |
| E2 | Neg | images/E2_Neg_6.JPG | 2.16 | 6.48 |
| E2 | Neg | images/E2_Neg_7.JPG | 2.16 | 6.44 |
| E2 | Neg | images/E2_Neg_8.JPG | 2.52 | 6.36 |
| E2 | Neg | images/E2_Neg_9.JPG | 2.48 | 6.12 |
| E2 | Neg | images/E2_Neg_10.JPG | 1.92 | 6.04 |
| E2 | Neg | images/E2_Neg_11.JPG | 2.56 | 5.92 |
| E2 | Neg | images/E2_Neg_12.JPG | 2.36 | 5.92 |
| E2 | Neg | images/E2_Neg_13.JPG | 2.56 | 5.64 |
| E2 | Neg | images/E2_Neg_14.JPG | 3.08 | 5.6 |
| E2 | Neu | images/E2_Neu_1.JPG | 5.24 | 3 |
| E2 | Neu | images/E2_Neu_2.JPG | 6 | 3 |
| E2 | Neu | images/E2_Neu_3.JPG | 5.76 | 2.68 |
| E2 | Neu | images/E2_Neu_4.JPG | 5.84 | 2.68 |
| E2 | Neu | images/E2_Neu_5.JPG | 5.72 | 2.64 |
| E2 | Neu | images/E2_Neu_6.JPG | 5.84 | 2.64 |
| E2 | Neu | images/E2_Neu_7.JPG | 5.64 | 2.6 |
| E2 | Neu | images/E2_Neu_8.JPG | 5.76 | 2.56 |
| E2 | Neu | images/E2_Neu_9.JPG | 5.92 | 2.52 |
| E2 | Neu | images/E2_Neu_10.JPG | 5.96 | 2.52 |
| E2 | Neu | images/E2_Neu_11.JPG | 5.6 | 2.2 |
| E2 | Neu | images/E2_Neu_12.JPG | 6 | 2.16 |
| E2 | Neu | images/E2_Neu_13.JPG | 5.76 | 2.04 |
| E2 | Neu | images/E2_Neu_14.JPG | 5.36 | 1.8 |

**Table S17. Mean Arousal and Valence ratings for each image set**

| **Set** | **Type of image** | **Valence (mean)** | **Arousal (mean)** |
| --- | --- | --- | --- |
| E1 | Neg | 2.15 | 6.37 |
| E2 | Neg | 2.15 | 6.36 |
| E1 | Neu | 5.72 | 2.48 |
| E2 | Neu | 5.74 | 2.50 |

**Table S18. Means valence and arousal ratings for negative and neutral images of the emotional reactivity task and their ID.**

| Negative images (IAPS) | | | Neutral images (MST) | | |
| --- | --- | --- | --- | --- | --- |
| ID | Val-M | Aro-M | ID | Val-M | Aro-M |
| 3000 | 1.48 | 7.48 | Set 6 025a | 5.88 | 3.04 |
| 3061 | 1.80 | 6.84 | Set 6 161a | 5.40 | 2.76 |
| 3069 | 1.64 | 6.80 | Set 2 014a | 5.96 | 2.72 |
| 3071 | 2.04 | 6.56 | Set 3 188b | 6.04 | 2.68 |
| 3170 | 2.04 | 6.60 | Set 3 176a | 5.64 | 2.64 |
| 3266 | 2.16 | 6.64 | Set 3 087a | 6.12 | 2.64 |
| 3015 | 2.08 | 6.52 | Set 3 020b | 5.44 | 2.60 |
| 3051 | 2.20 | 6.16 | Set 1 076b | 5.76 | 2.60 |
| 3064 | 2.12 | 6.12 | Set 2 023a | 5.96 | 2.56 |
| 3080 | 2.36 | 6.04 | Set 2 062a | 5.88 | 2.40 |
| 3131 | 3.12 | 6.00 | Set 2 185b | 5.48 | 2.24 |
| 3213 | 2.44 | 5.84 | Set 4 182b | 5.40 | 2.04 |
| 3261 | 2.28 | 5.96 | Set 5 125a | 5.60 | 2.04 |
| 3400 | 2.32 | 5.56 | Set 6 039b | 5.48 | 1.80 |
| 3016 | 1.36 | 7.36 | Set 3 116a | 5.24 | 3.00 |
| 3019 | 1.36 | 7.08 | Set 3 053a | 6.00 | 3.00 |
| 3053 | 1.64 | 6.76 | Set 3 030b | 5.76 | 2.68 |
| 3062 | 1.80 | 6.68 | Set 1 088b | 5.84 | 2.68 |
| 3063 | 2.16 | 6.60 | Set 1 090b | 5.72 | 2.64 |
| 3068 | 2.16 | 6.48 | Set 1 108b | 5.84 | 2.64 |
| 3100 | 2.16 | 6.44 | Set 2 118b | 5.64 | 2.60 |
| 3150 | 2.52 | 6.36 | Set 2 168b | 5.76 | 2.56 |
| 3060 | 2.48 | 6.12 | Set 2 178a | 5.92 | 2.52 |
| 3102 | 1.92 | 6.04 | Set 4 060a | 5.96 | 2.52 |
| 3120 | 2.56 | 5.92 | Set 4 055a | 5.60 | 2.20 |
| 3130 | 2.36 | 5.92 | Set D 137b | 6.00 | 2.16 |
| 3140 | 2.56 | 5.64 | Set 4 181a | 5.76 | 2.04 |
| 9405 | 3.08 | 5.60 | Set 5 143b | 5.36 | 1.80 |

**Table S19. Mean valence and arousal ratings for negative and neutral images of the emotional memory task and their ID.**

| Negative images | | | | | | Neutral images | | | | | |
| --- | --- | --- | --- | --- | --- | --- | --- | --- | --- | --- | --- |
| IAPS | | | NAPS | | | IAPS | | | NAPS | | |
| ID | Val-M | Aro-M | ID | Val-M | Aro-M | ID | Val-M | Aro-M | ID | Val-M | Aro-M |
| 2710 | 2.52 | 5.46 | A063h | 2.47 | 7.06 | 7006 | 4.88 | 2.33 | P104h | 5.38 | 4.12 |
| 3005.1 | 1.63 | 6.20 | A060h | 3.02 | 6.59 | 7950 | 4.94 | 2.28 | O314h | 4.93 | 4.70 |
| 6838 | 2.45 | 5.80 | F284h | 2.43 | 6.83 | 7179 | 5.06 | 2.88 | O313h | 5.46 | 4.41 |
| 6530 | 2.76 | 6.18 | F010h | 2.24 | 7.73 | 7044 | 4.69 | 3.94 | O224h | 4.89 | 4.91 |
| 6210 | 2.95 | 6.34 | L139h | 2.43 | 6.44 | 7595 | 4.55 | 3.77 | O179h | 4.85 | 4.98 |
| 8485 | 2.73 | 6.46 | O125h | 2.02 | 6.20 | 7248 | 5.22 | 4.22 | O245h | 5.20 | 4.88 |
| 9006 | 2.34 | 5.76 | P082h | 3.00 | 5.76 | 7018 | 4.81 | 3.91 | L020h | 4.65 | 5.02 |
| 9254 | 2.03 | 6.04 | P242v | 2.86 | 7.04 | 5740 | 5.21 | 2.59 | L084v | 5.40 | 4.69 |
| 9302 | 2.32 | 5.58 | P001h | 2.69 | 6.84 | 2396 | 4.91 | 3.34 | F218h | 4.96 | 4.85 |
| 9424 | 2.87 | 5.78 | A037h | 3.03 | 6.69 | 2749 | 5.04 | 3.76 | F055h | 5.42 | 4.90 |
| 9560 | 2.12 | 5.50 | A067h | 2.96 | 6.00 | 7247 | 5.05 | 4.14 | P159h | 5.44 | 4.43 |
| 2730 | 2.45 | 6.80 | F365v | 1.98 | 7.10 | 7830 | 5.26 | 4.08 | O161v | 5.44 | 4.48 |
| 2375.1 | 2.20 | 4.88 | F007h | 2.87 | 6.65 | 7017 | 5.18 | 3.12 | O146h | 4.48 | 4.88 |
| 6200 | 2.71 | 6.21 | O002h | 2.66 | 6.27 | 7000 | 5.00 | 2.42 | O089h | 5.09 | 4.93 |
| 6350 | 1.90 | 7.29 | O149h | 2.28 | 7.18 | 7632 | 5.22 | 4.78 | O234h | 4.43 | 4.97 |
| 6834 | 2.91 | 6.28 | P118h | 2.48 | 5.98 | 7705 | 4.77 | 2.65 | O282h | 4.36 | 5.09 |
| 9400 | 2.50 | 5.99 | P086h | 3.06 | 7.02 | 7011 | 4.52 | 3.81 | L093h | 5.41 | 4.56 |
| 9570 | 1.68 | 6.14 | P136h | 2.60 | 6.10 | 2411 | 5.07 | 2.86 | L111h | 5.48 | 4.13 |
| 9425 | 2.67 | 5.92 | A057h | 2.92 | 5.69 | 5534 | 4.84 | 3.14 | L044h | 5.13 | 4.46 |
| 9321 | 2.81 | 6.24 | A068h | 2.56 | 6.63 | 2393 | 4.87 | 2.93 | F306v | 5.41 | 4.80 |
| 9040 | 1.67 | 5.82 | A064v | 2.96 | 5.98 | 7043 | 5.17 | 3.68 | O296h | 5.15 | 4.98 |
| 9007 | 2.49 | 5.03 | F041h | 3.02 | 6.69 | 7920 | 4.51 | 3.87 | O276h | 5.41 | 4.55 |
| 2800 | 1.78 | 5.49 | L002h | 2.80 | 6.44 | 7062 | 5.27 | 3.40 | O247h | 5.17 | 4.81 |
| 3350 | 1.88 | 5.72 | O285h | 2.73 | 6.53 | 7031 | 4.52 | 2.03 | O108v | 4.83 | 5.00 |
| 6550 | 2.73 | 7.09 | P233h | 2.47 | 6.92 | 7547 | 5.21 | 3.18 | O246h | 4.90 | 4.73 |
| 6831 | 2.59 | 5.55 | P235h | 2.67 | 6.57 | 7186 | 4.63 | 3.60 | L057h | 5.48 | 5.25 |
| 6540 | 2.19 | 6.83 | P003h | 2.60 | 7.11 | 7100 | 5.24 | 2.89 | L056h | 4.82 | 4.77 |
| 9630 | 2.96 | 6.06 | A033h | 2.41 | 6.61 | 6150 | 5.08 | 3.22 | F276h | 5.48 | 4.90 |
| 9185 | 1.97 | 5.65 | A056h | 1.86 | 7.50 | 2410 | 4.62 | 4.13 | A093h | 5.38 | 4.72 |
| 9250 | 2.57 | 6.60 | F016h | 2.92 | 7.02 | 2026 | 4.82 | 3.40 | P149h | 5.49 | 4.21 |
| 9620 | 2.70 | 6.11 | F302h | 3.02 | 6.13 | 2383 | 4.72 | 3.41 | O152v | 5.19 | 5.10 |
| 9075 | 1.66 | 6.04 | O001h | 2.72 | 7.15 | 9210 | 4.53 | 3.08 | O187h | 4.73 | 4.96 |
| 9300 | 2.26 | 6.00 | O157h | 2.93 | 6.04 | 7040 | 4.69 | 2.69 | O168v | 5.51 | 5.00 |
| 2900 | 2.45 | 5.09 | P225h | 2.50 | 6.82 | 7249 | 5.24 | 3.97 | O191v | 5.23 | 4.63 |
| 3220 | 2.49 | 5.52 | P004h | 2.85 | 6.49 | 7038 | 4.82 | 3.01 | L159h | 4.70 | 4.88 |
| 6242 | 2.69 | 5.43 | P002v | 2.96 | 6.40 | 7004 | 5.04 | 2.00 | L091h | 5.16 | 4.57 |
| 6571 | 2.85 | 5.59 | A013h | 2.90 | 6.73 | 7211 | 4.81 | 4.20 | L024v | 4.80 | 4.36 |
| 6213 | 2.91 | 5.86 | A032h | 2.96 | 6.30 | 7491 | 4.82 | 2.39 | F201v | 5.30 | 4.78 |
| 9412 | 1.83 | 6.72 | F293h | 2.44 | 7.08 | 1675 | 5.24 | 4.37 | F309h | 5.25 | 4.84 |
| 9940 | 1.62 | 7.15 | F143v | 1.85 | 7.44 | 2595 | 4.88 | 3.71 | P146h | 4.94 | 4.90 |
| 9927 | 2.71 | 5.29 | O144h | 3.00 | 5.68 | 2484 | 5.00 | 3.75 | O306h | 5.41 | 4.78 |
| 9163 | 2.10 | 6.53 | O003h | 2.39 | 6.79 | 9260 | 4.63 | 3.45 | O299h | 5.08 | 4.92 |
| 9252 | 1.98 | 6.64 | P226h | 1.81 | 7.40 | 7242 | 5.28 | 3.83 | O244h | 5.07 | 4.98 |
| 9571 | 1.96 | 5.64 | P008h | 2.72 | 6.36 | 7590 | 4.75 | 3.80 | O134h | 4.32 | 4.84 |
| 2703 | 1.91 | 5.78 | P039v | 2.76 | 6.49 | 7020 | 4.97 | 2.17 | O162h | 5.44 | 4.98 |
| 2717 | 2.58 | 5.70 | A027h | 2.55 | 6.34 | 7003 | 5.00 | 3.07 | L016h | 5.13 | 4.09 |
| 6830 | 2.82 | 6.21 | A071h | 2.35 | 6.68 | 7217 | 4.82 | 2.43 | L060h | 5.21 | 4.27 |
| 6510 | 2.46 | 6.96 | A008v | 2.63 | 6.80 | 7550 | 5.27 | 3.95 | F198h | 4.72 | 4.91 |
| 8230 | 2.95 | 5.91 | F290h | 2.77 | 6.27 | 2487 | 5.20 | 4.05 | F192h | 5.43 | 4.78 |
| 9905 | 2.55 | 5.93 | L007h | 2.93 | 6.33 | 2215 | 4.63 | 3.38 | O115h | 4.74 | 5.04 |
| 9050 | 2.43 | 6.36 | O283h | 2.62 | 6.89 | 2102 | 5.16 | 3.03 | O112h | 4.97 | 4.53 |
| 9491 | 2.78 | 5.69 | P127h | 2.15 | 7.62 | 7235 | 4.96 | 2.83 | O311h | 5.04 | 4.98 |
| 9295 | 2.39 | 5.11 | P038h | 1.73 | 8.05 | 7497 | 5.19 | 4.97 | O211h | 5.09 | 4.87 |
| 9183 | 1.69 | 6.58 | P119h | 3.00 | 5.70 | 7002 | 4.97 | 3.16 | O068v | 5.24 | 4.80 |
| 9340 | 2.41 | 5.16 | A075h | 3.04 | 6.43 | 7175 | 4.87 | 1.72 | L019h | 5.18 | 4.31 |
| 2981 | 2.76 | 5.97 | A016h | 2.67 | 6.92 | 7150 | 4.72 | 2.61 | L036h | 5.20 | 4.88 |
| 2053 | 2.47 | 5.25 | A054h | 2.64 | 6.53 | 7025 | 4.63 | 2.71 | F215v | 4.67 | 4.85 |
| 6821 | 2.38 | 6.29 | F018h | 2.41 | 6.80 | 7021 | 5.21 | 4.17 | A129h | 5.22 | 5.02 |
| 6300 | 2.59 | 6.61 | L118v | 3.08 | 6.69 | 5535 | 4.81 | 4.11 | P150h | 5.21 | 4.64 |
| 6230 | 2.37 | 7.35 | O139h | 2.08 | 6.64 | 2745.1 | 5.31 | 3.26 | O310h | 5.28 | 4.66 |
| 9903 | 2.36 | 5.71 | P133h | 3.00 | 6.24 | 2308 | 5.22 | 3.82 | O308h | 5.02 | 4.83 |
| 9332 | 2.25 | 5.34 | P140h | 2.42 | 6.48 | 1616 | 5.21 | 3.95 | O130h | 4.98 | 4.69 |
| 9904 | 2.39 | 6.08 | P124h | 3.02 | 6.35 | 9468 | 4.67 | 4.68 | O279h | 5.43 | 4.88 |
| 9043 | 2.52 | 5.50 | A074h | 1.70 | 7.37 | 7077 | 5.12 | 4.61 | O204h | 5.13 | 4.63 |
| 9500 | 2.42 | 5.82 | A001h | 2.57 | 6.44 | 7019 | 5.20 | 3.36 | L067h | 4.98 | 4.76 |
| 9419 | 2.55 | 5.19 | F032h | 2.63 | 6.40 | 7010 | 4.94 | 1.76 | L015h | 4.59 | 4.94 |
| 2811 | 2.17 | 6.90 | F172h | 2.45 | 6.84 | 7185 | 4.97 | 2.64 | F220h | 4.73 | 4.80 |
| 3230 | 2.02 | 5.41 | O132h | 2.88 | 6.76 | 7160 | 5.02 | 3.07 | P097h | 5.27 | 4.80 |
| 6570 | 2.19 | 6.24 | O121v | 3.04 | 6.44 | 7255 | 5.07 | 3.36 | O071h | 4.98 | 5.06 |
| 6370 | 2.70 | 6.44 | P021h | 2.70 | 6.71 | 7180 | 4.73 | 3.43 | O312h | 5.16 | 4.89 |
| 6231 | 2.49 | 6.82 | P023h | 3.06 | 7.33 | 1350 | 5.25 | 4.37 | O196h | 5.09 | 4.96 |
| 9187 | 1.81 | 6.45 | P144h | 2.96 | 6.29 | 2214 | 5.01 | 3.46 | O222h | 4.73 | 5.02 |
| 9322 | 2.24 | 5.73 | A019h | 2.68 | 6.40 | 2850 | 5.22 | 3.00 | L081h | 5.48 | 4.90 |
| 9140 | 2.19 | 5.38 | A038h | 2.96 | 6.68 | 7037 | 4.81 | 3.71 | L077v | 4.64 | 4.65 |
| 9611 | 2.71 | 5.75 | A048h | 2.85 | 6.62 | 7016 | 4.76 | 3.40 | F311h | 5.43 | 4.76 |
| 9925 | 2.84 | 5.59 | F283h | 2.48 | 7.26 | 7030 | 4.69 | 2.99 | F312h | 5.04 | 4.63 |
| 9800 | 2.04 | 6.05 | L022h | 2.75 | 6.85 | 7161 | 4.98 | 2.98 | O172v | 5.31 | 4.86 |
| 2683 | 2.62 | 6.21 | O022h | 3.04 | 6.54 | 7184 | 4.84 | 3.66 | O210h | 4.85 | 4.63 |
| 3215 | 2.51 | 5.44 | P013v | 2.53 | 6.65 | 7009 | 4.93 | 3.01 | O277h | 4.59 | 4.98 |
| 6263 | 2.48 | 6.62 | P020h | 2.43 | 6.78 | 7170 | 5.14 | 3.21 | O208h | 5.13 | 4.92 |
| 6825 | 2.81 | 5.36 | P016h | 2.45 | 7.04 | 2514 | 5.19 | 3.50 | O251v | 4.83 | 4.74 |
| 6560 | 2.16 | 6.53 | A045h | 3.02 | 6.82 | 2397 | 4.98 | 2.77 | L013v | 4.35 | 5.07 |
| 9181 | 2.26 | 5.39 | A025h | 2.65 | 6.63 | 2702 | 5.21 | 3.92 | L078h | 4.50 | 4.86 |
| 9325 | 1.89 | 6.01 | F146h | 3.07 | 6.42 | 5531 | 5.15 | 3.69 | L063h | 5.49 | 4.63 |
| 9414 | 2.06 | 6.49 | F019h | 2.79 | 6.65 | 7207 | 5.15 | 3.57 | F216h | 4.91 | 4.85 |
| 9520 | 2.46 | 5.41 | L026h | 2.48 | 6.41 | 7187 | 5.07 | 2.30 | F194h | 5.23 | 4.90 |
| 9810 | 2.09 | 6.62 | O011h | 2.87 | 5.89 | 7090 | 5.19 | 2.61 | A105h | 5.35 | 4.77 |
| 9922 | 2.78 | 5.21 | P200h | 2.27 | 6.93 | 7050 | 4.93 | 2.75 | P091h | 5.26 | 5.04 |
| 2345.1 | 2.26 | 5.50 | P009h | 2.70 | 6.51 | 7041 | 4.99 | 2.60 | O237h | 5.50 | 4.27 |
| 2688 | 2.73 | 5.98 | P128h | 2.23 | 7.75 | 7487 | 4.92 | 4.08 | O186v | 4.55 | 4.83 |
| 6563 | 1.77 | 6.85 | A077h | 2.02 | 7.48 | 5510 | 5.15 | 2.82 | O239v | 5.00 | 4.74 |
| 6212 | 2.19 | 6.01 | A042h | 2.82 | 6.32 | 2381 | 5.25 | 3.04 | O189h | 5.07 | 4.93 |
| 7380 | 2.46 | 5.88 | F153v | 2.65 | 6.83 | 2377 | 5.19 | 3.50 | O231h | 5.21 | 4.64 |
| 9413 | 1.76 | 6.81 | F028h | 2.75 | 6.72 | 7476 | 4.99 | 4.63 | L043h | 4.80 | 5.11 |
| 9184 | 2.47 | 5.75 | O004h | 3.05 | 6.47 | 7287 | 4.77 | 3.57 | L061h | 4.98 | 4.59 |
| 9326 | 2.21 | 5.89 | P201v | 1.87 | 7.51 | 7045 | 4.97 | 3.32 | F180h | 5.27 | 4.67 |
| 9530 | 2.93 | 5.20 | P017h | 2.96 | 7.44 | 7034 | 4.95 | 3.06 | P078v | 4.98 | 5.09 |
| 9941 | 2.91 | 5.83 | P125h | 2.69 | 6.14 | 7032 | 4.82 | 3.18 | O307v | 5.07 | 4.79 |
| 9421 | 2.21 | 5.04 | P022h | 2.13 | 6.48 | 7110 | 4.55 | 2.27 | O280v | 4.82 | 5.07 |
| 2751 | 2.67 | 5.18 | A207h | 3.06 | 6.69 | 7055 | 4.90 | 3.02 | O230h | 5.32 | 5.07 |
| 3180 | 1.92 | 5.77 | A078h | 2.26 | 6.68 | 5471 | 5.21 | 3.26 | O223h | 5.20 | 4.82 |
| 6260 | 2.44 | 6.93 | A024h | 2.44 | 6.75 | 2880 | 5.18 | 2.96 | O175h | 4.43 | 5.00 |
| 6360 | 2.23 | 6.33 | F031v | 2.79 | 6.81 | 2385 | 5.20 | 3.64 | L080h | 5.46 | 4.35 |
| 6415 | 2.21 | 6.20 | F152h | 2.71 | 6.60 | 1908 | 5.28 | 4.88 | L079v | 5.09 | 4.76 |
| 9301 | 2.26 | 5.28 | O007h | 2.98 | 6.24 | 8121 | 4.63 | 4.14 | F211h | 5.48 | 5.06 |
| 9429 | 2.68 | 5.63 | P075v | 2.75 | 6.63 | 7036 | 4.88 | 3.32 | O185h | 5.18 | 5.04 |
| 9908 | 2.34 | 6.63 | P142h | 2.85 | 6.04 | 7035 | 4.98 | 2.66 | O226h | 4.98 | 5.00 |
| 9427 | 2.89 | 5.50 | P143h | 2.57 | 6.19 | 7484 | 4.99 | 4.24 | O281h | 4.56 | 4.96 |
| 9635.1 | 1.90 | 6.54 |  |  |  | 7130 | 4.77 | 3.35 | O278h | 5.48 | 4.49 |
| 9900 | 2.46 | 5.58 |  |  |  | 7012 | 4.98 | 3.00 | O176v | 5.15 | 5.00 |
| 2799 | 2.42 | 5.02 |  |  |  | 7056 | 5.07 | 3.07 | L076h | 5.06 | 4.58 |
| 6313 | 1.98 | 6.94 |  |  |  | 7080 | 5.27 | 2.32 | L012h | 4.40 | 4.62 |
| 6520 | 1.94 | 6.59 |  |  |  | 2446 | 4.70 | 3.79 | F320v | 5.06 | 4.98 |
| 6243 | 2.33 | 5.99 |  |  |  | 2191 | 5.30 | 3.61 |  |  |  |
| 7359 | 2.92 | 5.36 |  |  |  | 2038 | 5.09 | 2.94 |  |  |  |
| 9920 | 2.50 | 5.76 |  |  |  | 9700 | 4.77 | 3.21 |  |  |  |
| 9921 | 2.04 | 6.52 |  |  |  | 7059 | 4.93 | 2.73 |  |  |  |
| 9428 | 2.31 | 5.66 |  |  |  | 7182 | 5.16 | 4.02 |  |  |  |
| 9610 | 2.89 | 5.23 |  |  |  | 7233 | 5.09 | 2.77 |  |  |  |
| 9430 | 2.63 | 5.26 |  |  |  | 7058 | 5.29 | 3.98 |  |  |  |
| 9909 | 2.78 | 5.98 |  |  |  | 7365 | 5.20 | 4.13 |  |  |  |
| 2095 | 1.79 | 5.25 |  |  |  | 7014 | 5.15 | 3.25 |  |  |  |
| 3500 | 2.21 | 6.99 |  |  |  | 7053 | 5.22 | 2.95 |  |  |  |
| 6312 | 2.48 | 6.37 |  |  |  | 5532 | 5.19 | 3.79 |  |  |  |
| 6250 | 2.83 | 6.54 |  |  |  | 2890 | 4.95 | 2.95 |  |  |  |
| 6315 | 2.31 | 6.38 |  |  |  | 2309 | 4.89 | 4.33 |  |  |  |
| 9423 | 2.61 | 5.66 |  |  |  | 2279 | 4.71 | 3.74 |  |  |  |
| 9600 | 2.48 | 6.46 |  |  |  |  |  |  |  |  |  |
| 9910 | 2.06 | 6.20 |  |  |  |  |  |  |  |  |  |
| 9911 | 2.30 | 5.76 |  |  |  |  |  |  |  |  |  |
| 9901 | 2.27 | 5.70 |  |  |  |  |  |  |  |  |  |
| 9902 | 2.33 | 6.00 |  |  |  |  |  |  |  |  |  |

**Note:** We simplified the ID of the NAPS images to allow appropriate table dimensions. Then, readers could use the following abbreviations to return to the original ID: A = Animals, F = Faces, L = Landscapes, O = Objects, P = People.

Table S20. **Contingency table of picture-set assignment across groups**

|  | E1 | E2-E1 | E1-E2 | E2 |
| --- | --- | --- | --- | --- |
| No-REM | 4 | 8 | 5 | 3 |
| REM | 4 | 3 | 4 | 9 |
| Wake | 10 | 10 | 10 | 10 |
